# An Overview of Bioproducts from Wastewater-Grown Microalgae: Recent Advancements, Economic and Feasibility Concerns

**DOI:** 10.3390/microorganisms14071494

**Published:** 2026-07-08

**Authors:** Alexandru Vlaicu, Ana-Maria Surupăceanu, Alin Cristian Nicolae Vintilă, Andreea Luiza Mîrț, Mihaela Cîlțea-Udrescu, Anca Paulenco, Gabriel Vasilievici

**Affiliations:** 1 National Institute for Research and Development in Chemistry and Petrochemistry—ICECHIM, Spl. Independentei Nr. 202, Sector 6, 060021 Bucharest, Romania; alexandru.vlaicu@icechim.ro (A.V.); anamaria.surupaceanu@icechim.ro (A.-M.S.); mihaela.ciltea@icechim.ro (M.C.-U.); anca.paulenco@icechim.ro (A.P.); gvasilievici@icechim.ro (G.V.); 2 Department of Science and Engineering of Oxide Materials and Nanomaterials, Faculty of Chemical Engineering and Biotechnologies, National University of Science and Technology Politehnica Bucharest, 060042 Bucharest, Romania; 3 Department of Bioresources and Polymer Science, Faculty of Chemical Engineering and Biotechnologies, National University of Science and Technology Politehnica Bucharest, 060042 Bucharest, Romania

**Keywords:** phycoremediation, microalgal biorefinery, wastewater treatment, techno-economic analysis, life cycle assessment

## Abstract

Global wastewater generated in unprecedented volumes places a significant strain on aquatic environments, challenging the municipal sector to transition from energy-heavy pollutant removal technologies towards alternative resource recovery strategies with a low carbon footprint. The aim of this work is to review microalgae-based phycoremediation as a sustainable, cost-effective alternative that extracts nutrients from municipal, agricultural, and industrial streams without generating secondary chemical pollution. By utilizing these nutrient-rich effluents, microalgal cultivation generates valuable biomass for downstream valorization into biofuels, biofertilizers, biostimulants, and high-value biopolymers, while eliminating or reducing the costs of synthetic cultivation mediums. However, integrated techno-economic analyses (TEA) and life cycle assessments (LCA) reveal critical socio-technical bottlenecks.

## 1. Introduction

Unprecedented global wastewater volumes—fueled by population growth, urbanization, and industrialization—now place an unsustainable strain on natural aquatic environments. Globally, freshwater is heavily skewed toward production sectors, with agriculture accounting for approximately 70% of total usage and industry consuming 20%, leaving a mere 10% for domestic household consumption. Despite this massive extraction infrastructure, global water equity remains unachieved, as nearly 2.2 billion individuals still lack access to safely managed drinking water service [1]. While conventional wastewater treatment initially focused on safeguarding human health, contemporary environmental frameworks have mandated an additional focus on ecological protection via nutrient pollution control. This shift drove the widespread implementation of advanced nitrogen (N) and phosphorus (P) removal technologies within wastewater treatment plants (WWTPs). Despite their operational efficacy, these systems carry a heavy sustainability deficit. They are notoriously energy-intensive, rely heavily on chemical additives like metal salts for chemical phosphorus precipitation, incur a significant carbon footprint, and produce vast quantities of secondary sludge that present severe techno-economic challenges for disposal [2,3]. Traditional wastewater treatment plants, which mostly depend on activated sludge and chemical treatments, are struggling to meet today’s stricter environmental standards [4]. As a result, the modern wastewater sector must transition from energy-heavy pollutant removal to low-carbon technologies that focus on recovering resources [5].

Microalgae systems can emit non-negligible amounts of nitrous oxide (N_2_O)—a gas with a global warming potential over 300 times that of CO_2_. Previous research attributes N_2_O emissions in microalgae systems to indirect bacterial pathways—specifically, microalgae acting as a carbon source for denitrification, photosynthetic oxygen suppressing complete denitrification, nitrite accumulation, or carbon competition inhibiting nitrifiers. In contrast, very few studies investigate direct N_2_O production by the microalgae themselves, and its specific mechanisms within wastewater treatment remain largely unexplored. Because microalgal N_2_O production relies on a sequence of redox reactions, utilizing partially reduced nitrogen species like NO_2_^−^ or NO significantly enhances generation by bypassing upstream enzymatic reduction steps. In real wastewater treatment scenarios, N_2_O emissions increased substantially in streams with elevated NO_2_^−^ fractions, whereas emissions were minimized when NH_4_^+^ was the dominant pollutant [6]. These dynamics demonstrate that direct microalgal N_2_O generation represents a significant environmental bottleneck that must be integrated into life cycle assessments of microalgae-based systems treating nitrate- or nitrite-rich wastewater streams [7,8]. Despite this disadvantage, phycoremediation presents a sustainable, cost-effective alternative by utilizing microalgae to naturally extract nitrogen (N), phosphorus (P), and heavy metals directly from wastewater. This approach prevents aquatic eutrophication without generating secondary chemical pollution [9]. Furthermore, compared to conventional methods, this process requires significantly less energy while generating valuable biomass that can be converted into bioproducts such as biofuels, biofertilizers, or bioplastics [10,11]. Consequently, integrating phycoremediation into a microalgal biorefinery offers an economically and environmentally viable path toward sustainability. By operating within a circular loop, this strategy maximizes resource recovery to achieve a true zero-waste process flow. This approach eliminates the prohibitive costs of utilizing clean freshwater and synthetic media for industrial microalgal cultivation, thereby rendering downstream biomass valorization economically feasible at scale [12].

## 2. Principles of Microalgae-Based Wastewater Treatment

**Figure 1:**
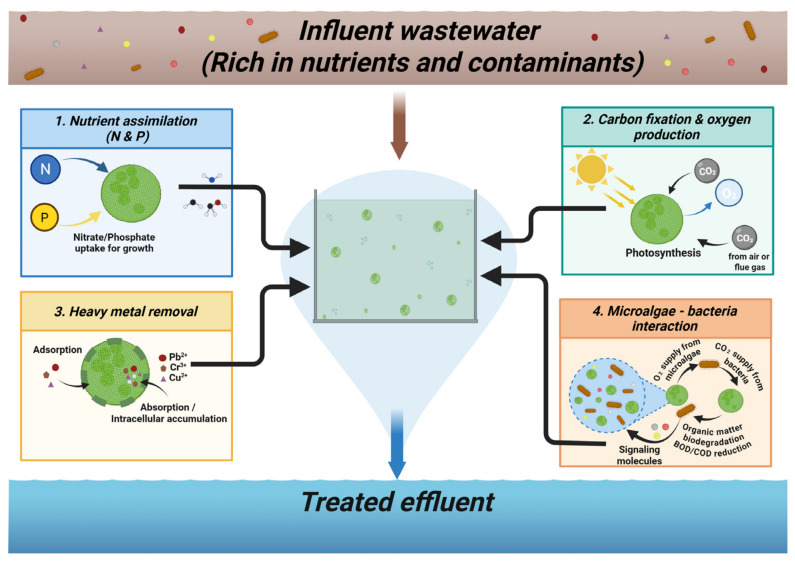
Wastewater remediation mechanism (created in BioRender, Udrescu M. 2026).

The future of wastewater (WW) treatment lies in sustainability and resource recovery, with current studies aiming for a 95% nutrient recovery rate. Microalgae meet this challenge perfectly, serving as a powerful, green alternative to traditional treatment systems (Figure 1). Wastewater is rich in valuable nutrients suitable for microalgae growth; utilizing these streams for cultivation significantly enhances the overall sustainability of the process. Wastewater streams are frequently laden with toxic compounds and high concentrations of organic and inorganic nutrients, reflected in elevated COD and BOD levels. When released into the environment, excessive nitrogen and phosphorus inputs trigger eutrophication. This ecological imbalance leads to severe environmental degradation, including the generation of solid wastes and unpleasant atmospheric emissions [13].

Table 1 presents an overview of various microalgal species, cyanobacteria, and symbiotic consortia evaluated across municipal, agricultural, and industrial wastewater streams, highlighting their specific characteristics. Although each of these effluents presents severe environmental risks if discharged untreated, they vary substantially in their chemical compositions, operational demands, and underlying mechanisms of microalgae interaction. Municipal wastewater is predominantly defined by its highly biodegradable organic fraction. Conversely, agricultural effluents—particularly dairy and piggery streams—exhibit substantially higher, more concentrated macronutrient loads embedded within complex matrices of lactose, lipids, and fats [14]. A critical distinction lies in the nature of their chemical threats; whereas municipal sewage typically contains trace “micropollutants” (pharmaceuticals and hormones in the microgram range), poultry and industrial effluents pose severe, acute toxicity challenges. These streams are characterized by high concentrations of antibiotics, heavy metals, and recalcitrant or highly colored industrial agents like textile dyes [15]. Wastewater derived from fish processing operations introduces unique physical constraints due to high concentrations of suspended oils and grease. Consequently, these effluents require preliminary physicochemical treatment—specifically fat and oil separation—before microalgae can be effectively deployed as a final polishing stage. This stands in contrast to streams like municipal wastewater, which generally permit more direct biological processing [16].

### 2.1. Wastewater Streams

#### 2.1.1. Municipal Wastewater

As the human population grows, total municipal wastewater production increases proportionally. Because municipal wastewater is primarily composed of residential water use, its contamination is characterized chiefly by easily degradable organic matter. However, several persistent organic pollutants also occur in micro-concentrations below the mg L^−1^ range. Often referred to as “micropollutants,” these trace chemicals primarily include pharmaceuticals, hormones, surfactants, plasticizers, flame retardants, and pesticides [17].

Typically, conventional municipal wastewater treatment follows a five-stage system: preliminary, primary, secondary, tertiary or advanced treatment, and sludge management. These consecutive stages utilize a combination of physical, chemical, and biological processes to eliminate solids, organic matter, nutrients, and other contaminants. Depending on the specific composition of the influent, supplementary treatment steps may be required at the beginning or end of the process [18]. While municipal wastewater typically contains high levels of nitrogen, phosphorus, and organic carbon, it also harbors a diverse array of contaminants—such as pharmaceuticals and heavy metals—introduced via stormwater runoff and extraneous water inflows. Despite the presence of these trace pollutants, treatment primarily focuses on removing organic matter, nitrogen, and phosphorus, as these macronutrients are the leading drivers of eutrophication in receiving water bodies [19].

Recent studies on microalgae-based municipal wastewater treatment demonstrate highly effective nitrogen and phosphorus removal. Furthermore, this approach offers additional environmental benefits, including increased dissolved oxygen levels, reduced bacterial loads, and the removal of heavy metals.

Purba et al. [20] demonstrated that cultivation of *Chlorella sorokiniana* in municipal wastewater samples had higher biomass production of 0.825 g L^−1^ and reduced environmental impacts compared with AF-6 medium, lowering the global warming potential GWP from 6.773 to 5.305 kg CO_2_ eq. Van et al. [21] achieved over 99% removal of COD, BOD, nitrogen, and phosphorus from municipal wastewater using *S. salina* M8. Similarly, Xu et al. developed a lignocellulosic carrier-based microalgal biofilm system using *Chlorella vulgaris*, *Scenedesmus quadricauda*, and their co-culture to remove nutrients from municipal wastewater. They achieve a rapid TN and TP removal, with efficiencies reaching 83.6% and 87.7%, respectively [13,22].

#### 2.1.2. Agricultural Wastewater

As the largest consumer of freshwater resources, agriculture accounts for approximately 70% of global water withdrawals. Furthermore, conventional agricultural practices—specifically the intensive application of fertilizers, pesticides, and herbicides—serve as major drivers of water pollution, ultimately degrading aquatic ecosystems and posing significant risks to human health. Consequently, the efficient treatment of agricultural wastewater is essential not only for safeguarding public health and the environment but also for preserving vital freshwater resources [23].

The concentration of organic matter is notably high, particularly regarding nitrogen and phosphorus, which can readily trigger freshwater eutrophication and broader environmental degradation. Conventional wastewater treatment technologies, although widely implemented, face several inherent limitations. These include high operational and energy costs, incomplete nutrient removal, secondary pollution from chemical additives, and the irreversible loss of valuable resources like nitrogen and phosphorus. Fortunately, microalgae can efficiently remove total nitrogen (TN), total phosphorus (TP), and chemical oxygen demand (COD) from aquaculture wastewater [24,25].

##### Dairy Wastewater

Globally, annual milk production reaches approximately 900 million tons, with processing operations generating roughly 2.5 times more wastewater than the actual volume of milk processed [26]. Dairy wastewater comprises a complex matrix of organic and inorganic constituents, notably carbohydrates (primarily lactose), proteins, lipids, fats, minerals, and macronutrients such as nitrogen and phosphorus compounds. The specific characteristics of this effluent vary widely depending on the processing methods, the types of dairy products manufactured, and the cleaning protocols employed [27].

Recent studies have increasingly utilized microalgae-based technologies for the treatment of dairy wastewater. Paulenco et al. showed that the microalgae Nannochloris sp. effectively eliminated 99–100% of lactose from the growth medium, while reducing chemical oxygen demand (COD), nitrogen, and phosphorus by up to 96%, 91%, and 70%, respectively [28]. Using *Porphyridium purpureum*, the same research group achieved complete lactose elimination, up to 92% COD removal, and 100% reduction for both TN and TP [29]. Phyu et al. evaluated monoculture, co-culture, and sequential culture strategies for dairy wastewater treatment utilizing two wastewater-borne cyanobacteria and two laboratory-cultured microalgae. Their findings revealed that sequential cultivation demonstrated superior performance, achieving the highest nutrient removal efficiencies: 90.5 ± 3.2% for NH_4_^+^-N, 86.6 ± 0.3% for TN, 78.8 ± 1.2% for COD, 74.8 ± 5.9% for TP [30]. Xiao et al. cultivated *Chlorella protothecoides* and *Chlamydomonas reinhardtii* in filtered livestock wastewater (LWW), yielding substantial removal of dissolved total nitrogen (86.8–95.3%), dissolved organic nitrogen (75.5–92.0%), NH_4_^+^-N (98.3–99.8%), and total phosphorus (57.2–100%). However, removal was moderate for dissolved organic carbon (12.1–67.5%) and 19 metal(loid)s (2.49–33.4% for total) [31]. In another study, Zhang et al. demonstrated that *Nodosilinea* sp. E11 is a promising candidate for dairy wastewater purification, achieving removal efficiencies of 56.60% for TN, 70.76% for TP, and 91.40% for NH_4_^+^-N [32]. Mahdavi et al. developed a co-cultivation system using *Tetraselmis chuii* and *Chlorella vulgaris* for dairy wastewater treatment. Utilizing a 3:1 ratio of *C. vulgaris* to *T. chuii*, they achieved over 90% reduction in nitrate and ammonium, alongside approximately 60% removal of COD and phosphates [33]. Cruz et al. evaluated the treatment of dairy effluent using *Chlorella fusca* LEB 111 and *Spirulina* sp. LEB 18. The respective removal efficiencies for *C. fusca* and *Spirulina* sp. were 98.5% and 99.1% for BOD, 96.5% and 97.7% for COD, 98.8% and 85.3% for total phosphorus, and 98% and 99% for ammonia nitrogen [34].

##### Fish Processing Wastewater

Wastewater from fish processing facilities is characterized by high concentrations of oil, grease, and nutrients. When discharged untreated, the constituent ammonia, nitrates, and phosphates can induce severe environmental degradation, including aquatic eutrophication, the destruction of natural coastal ecologies, and significant biodiversity loss [35]. To mitigate environmental degradation, fish processing effluent must be treated prior to discharge. Standard treatment configurations typically employ physicochemical processes as primary units to remove oils and particulate matter, followed by biological reactors—such as activated sludge oxidation basins—to degrade dissolved organic matter [36]. Several studies have assessed microalgae-based systems for treating fish processing wastewater.

Khalatbari et al. deployed *Chlorella sorokiniana* as a final polishing step for fish processing wastewater after fat and oil separation. This microalgal treatment significantly enhanced effluent quality, yielding final concentrations of approximately 6 mg L^−1^ for TN, ~100 mg L^−1^ for COD, and ~0.3 mg L^−1^ for TP [37]. Christodoulou et al. developed an innovative, modular photobioreactor (PBR) designed for simultaneous microalgae cultivation and aquaculture wastewater treatment. A microalgal consortium composed of *Chlorella* sp., *Scenedesmus* sp., and *Phormidium* sp. was evaluated for its growth potential and remediation efficiency. The system demonstrated high nutrient removal capabilities with 92% reduction in phosphate (removal rate: 0.07 mg L^−1^ d^−1^) and a 62% reduction in nitrate (removal rate: 1.1 mg L^−1^ d^−1^) [38]. Zhao et al. investigated the influence of nanoscale zero-valent iron (nZVI) on microalgae-based systems for the elimination of nutrients and antibiotics from aquaculture wastewater. Their results indicated that an nZVI concentration of 10 mg L^−1^ optimally promoted microalgal growth, with a co-culture of *Chlorella vulgaris*, endophytic bacteria, and the fungus *Clonostachys rosea* demonstrating the highest performance. This system achieved remarkable removal efficiencies, reaching 87.± 8.04% for COD, 87.76 ± 8.32% for TN, 88.12 ± 8.45% for TP, and 99.17 ± 0.52% for tetracycline [39]. Bulynina et al. investigated the cultivation of various algal strains (*Chlorella vulgaris* SB-M4, *Chlorella* sp. EE-P5, *Micractinium inermum* EE-M2, and *Tetradesmus obliquus* EZ-B11) in unsterilized wastewater from a fish processing plant. Their results demonstrated that ammonium, phosphate, and sulfate ions were completely removed from the effluent during microalgal growth [16].

##### Piggery Wastewater

Swine (or piggery) wastewater (SWW) is heavily enriched with macronutrients and organic matter. Specifically, it contains high loads of nitrogen (primarily ammonia nitrogen), phosphorus, and organic carbon, represented by its chemical oxygen demand (COD) and biochemical oxygen demand (BOD) [40]. Developing effective methods to treat PWW is a critical global challenge. Current remediation approaches integrate physical methods (filtration, grit chambers, flotation), chemical treatments (flocculation, oxidation, disinfection), and biological frameworks, including activated sludge (AS), anaerobic digestion (AD), and constructed wetlands (CWs). Biological wastewater treatment driven by microalgae has attracted significant attention as an inherently sustainable and environmentally friendly technology [41].

An ammonia-tolerant microalgal strain was isolated and identified as *Chlorella sorokiniana*. With this strain, the removal efficiencies of chemical oxygen demand, dissolved organics, total nitrogen, NH_3_-N, and total phosphorus significantly improved from 42%, 80%, 23%, 28%, and 34% to 70%, 88%, 66%, 72%, and 99%, respectively [42]. Four native microalgal strains present in piggery wastewater were isolated and molecularly identified as *Radiococcus polycoccus*, *Chlorolobion braunii*, *Micractinium* sp., and *Desmodesmus multivariabilis*. All strains demonstrated a strong capacity for removing nitrogen compounds from synthetic effluents, showing a clear preference for ammonium over nitrate assimilation [43]. Cultivation of the novel mixotrophic *Chlorella sorokiniana* Cbeo under indoor and outdoor conditions yielded high removal efficiencies for COD (91.9–96.7%), NH_4_^+^-N (96.6–99.7%), TN (96.2–96.4%), and TP (98.2–100%) [44]. An outdoor, pilot-scale, algal–bacterial raceway pond was operated in continuous mode for 208 days to treat piggery wastewater. The system achieved removal efficiencies of 90% for NH_4_^+^, 90% for PO_4_^3−^, and 59% for COD. Initially, the microalgal community was dominated by *Chlorellaceae* (1 × 10^6^ cell mL^−1^), accompanied by minor populations of *Scenedesmaceae* (0.2 × 10^6^ cell mL^−1^) and *Chlamydomonadaceae* (0.2 × 10^6^ cell mL^−1^) [45].

##### Poultry Wastewater

Untreated poultry industry wastewater contains high concentrations of lipids, proteins, blood, heavy metals, and antibiotics, posing a severe threat to river and creek ecosystems. Currently, conventional treatment methods fail to effectively neutralize these complex livestock effluents, meaning they are frequently discharged into waterways without adequate remediation [46]. A recent study by Wang et al. highlights algal–bacterial symbiosis as a highly effective approach for removing complex pollutants. The authors showed that heavy metal ion removal was mainly driven by adsorption via *Chlorella*, whereas antibiotic degradation was primarily mediated by bacteria [46]. The microalga *Neochloris* sp. also demonstrated efficient removal of COD, nitrite, and phosphate from poultry slaughterhouse waste [47]. Khiewwijit et al. successfully developed a high-performance biofilm microalgal system, achieving average removal efficiencies of 97% for NH_4_^+^-N, 93% for PO_4_^3−^-P, and 75% for dissolved COD. In comparison, their suspended microalgae system yielded slightly lower removal efficiencies of 92%, 87%, and 68% for the same parameters, respectively [48]. The performance of *Tetraselmis suecica* and *Micractinium reisseri* in treating poultry abattoir wastewater (25–100% concentrations) was investigated under controlled laboratory conditions. *T. suecica* outperformed *M. reisseri*, achieving higher removal efficiencies for BOD (94.3% vs. 84.7%), COD (94.5% vs. 86.2%), nitrate (98% vs. 95.4%), and phosphate (79.9% vs. 64.6%) [49].

#### 2.1.3. Industrial Wastewater

The primary sources of manufacturing wastewater include the petrochemical, pharmaceutical, pulp and paper, textile, iron and steel, and food processing industries. Regardless of the sector, industrial wastewater is generally categorized into three distinct streams: cooling water, washing water, and process wastewater [50]. Treating industrial effluents is inherently challenging due to their high organic loads, complex pollutant profiles, and poorly biodegradable components. However, microalgae represent a promising alternative within biological treatment, as they effectively assimilate these nutrients and convert them into valuable biomass [51]. Javed et al. evaluated the cultivation of *Chlorella vulgaris* in unsterilized, real textile wastewater, finding that a 50% dilution yielded the highest decolorization efficiency and COD removal [52]. Integrating *C. vulgaris* cultivation with plasmolysis for industrial wastewater treatment significantly enhances the remediation process, leading to substantially higher COD, TN, and TP removal efficiencies [53].

**Table 1:** Comparative overview of removal efficiencies when treating wastewater streams by various microalgae species.

Wastewater Category	Specific Stream	Characteristics	Microalgae Strains	Remediation Performance and Removal Efficiencies
Municipal	Municipal Sewage	Easily degradable organic matter; high N, P, and organic C; trace micropollutants (pharmaceuticals, hormones, pesticides).	*Chlorella sorokiniana* [20]	Biomass yield: 0.825 g L^−1^ (reduced GWP from 6.77 to 5.31 kg CO_2_ eq)
			*Synechocystis salina* M8 [21]	>99% COD, BOD, N, and P
			*Chlorella vulgaris* and *Scenedesmus quadricauda* (co-culture biofilm) [22]	83.6% TN 87.7% TP
Agricultural/Livestock	Dairy Wastewater	Complex matrix: lactose, proteins, lipids, fats, minerals, high N and P.	*Nannochloris* sp. [28]	>99% Lactose 96% COD 91% N 70% P
			*Porphyridium purpureum* [29]	>99% Lactose 92% COD 100% N 100% P
			*Tetraselmis chuii* and *C. vulgaris* (3:1 co-culture) [33]	>90 Nitrate and Ammonium 60% COD and Phosphates
			*Chlorella fusca* LEB 111 [34]	98.5% BOD 96.5% COD 98.8% TP 98% Ammonia-N
			*Spirulina* sp. LEB 18 [34]	99.1% BOD 97.7% COD 85.3% TP 99% Ammonia-N
	Piggery Wastewater (SWW)	Heavily enriched with macronutrients; high organic carbon, P, and N (primarily ammonia nitrogen).	*Chlorella sorokiniana* (Ammonia-tolerant strain) [42]	70% COD 72% Ammonia-N 66% TN 99% TP
			*Chlorella sorokiniana* Cbeo (Mixotrophic) [44]	91.9–96.7% COD 96.6–99.7% Ammonium-N 96.2–96.4% TN 98.2–100% TP
			Algal–bacterial consortium (*Chlorellaceae*-dominated, in a pilot raceway pond) [45]	59% COD 90% Ammonium-N 90% phosphate
	Poultry Wastewater	High lipids, proteins, blood, heavy metals, and antibiotics. Dangerous to river ecosystems.	Microalgae polyculture biofilm (dominated by *Chlorella* sp. and *Scenedesmus* sp.) [48]	75% COD 97% Ammonium-N 93% Phosphate
			*Tetraselmis suecica* [49]	94.3% BOD 94.5% COD 98% Nitrate 79.9% Phosphate
			*Micractinium reisseri* [49]	84.7% BOD 86.2% COD 95.4% Nitrate 64.6% Phosphate
	Fish Processing Wastewater	High concentrations of oils, grease, ammonia, nitrates, and phosphates.	*Chlorella sorokiniana* [37]	Residual effluent quality: COD ≈ 100 mg L^−1^ TN ≈ 6 mg L^−1^ TP ≈ 0.03 mg L^−1^
			Consortium (*Chlorella* sp., *Scenedesmus* sp., *Phormidium* sp.) [38]	92% Phosphate 62% Nitrate
			*C. vulgaris* + endophytic bacteria + fungus *Clonostachys rosea* (10 mg L^−1^ nZVI) [39]	87 ± 8.04% COD 87.76 ± 8.32% TN 88.12 ± 8.45% TP 99.17 ± 0.52% Tetracycline
Industrial	Textiles, Petrochemicals, Paper, etc.	High organic loads, complex pollutant profiles, and poorly biodegradable/toxic components (e.g., dyes).	*Chlorella vulgaris* (in real, unsterilized textile wastewater) [52])	50% dilution yielded 82% COD removal and the highest decolorization.
			*Chlorella vulgaris* (integrated with plasmolysis) [53]	Textile wastewater (TWW) 45 ± 3% COD 60 ± 5% TN 42 ± 1.8% TP Plasma-treated TWW 72 ± 5% COD 92 ± 3.5% TN 88 ± 2.7% TP

### 2.2. Physiological Traits and Biological Mechanisms Driving Microalgae-Based Waste Water Treatment

#### 2.2.1. Key Physiological Adaptations

Several key traits position microalgae as ideal candidates for biotechnological exploitation. Foremost among these are their capacity for rapid cellular division and biomass accumulation under optimized conditions, alongside their extensive biodiversity and robust metabolic adaptability across diverse environmental conditions [54].

• Rapid Cellular Division and Biomass Accumulation

This rapid growth rate drives highly efficient nutrient assimilation, allowing microalgae to convert inorganic pollutants, such as nitrogen and phosphorus, into valuable biomass within days. Consequently, this swift biological process effectively prevents eutrophication, reduces carbon footprints, and facilitates circular resource recovery [55,56]. Ma et al. reported a volumetric biomass yield of 0.0229 g L^−1^. Concurrently, the culture demonstrated robust remediation capacity, achieving a macronutrient removal efficiency exceeding 85% [57]. An analysis of *Chlorella vulgaris* cultivation relative to N:P ratios and nitrogen sources revealed that the strain grew successfully in all synthetic media. An N:P ratio of nine yielded the highest specific growth rate (0.74 ± 0.07 d^−1^), a trend that remained independent of the nitrogen source [58]. Optimizing the kinetic growth rates of *Chlamydomonas* species within wastewater treatment systems drives high-velocity nutrient assimilation, achieving up to 99% extraction efficiency for both reactive nitrogen and phosphorus while concurrently generating valuable cellular biomass. Under ideal cultivation regimes, target strains such as *Chlamydomonas reinhardtii* exhibit maximum specific growth rates ranging from 1.5 to 1.9 d^−1^, which translates to an exceptional volumetric biomass productivity of up to 2.0 g L^−1^ of dry weight [14,59].

• Extensive biodiversity

A wide array of microalgal and cyanobacterial genera—most notably *Scenedesmus*, *Chlorella*, *Botryococcus*, *Phormidium*, *Limnospira* (formerly *Arthrospira*/*Spirulina*), *Chlamydomonas*, *Euglena*, *Oscillatoria*, and *Ankistrodesmus*—have demonstrated exceptional robust growth and adaptation within complex wastewater matrices. These organisms display high-efficiency bioremediation pathways capable of concurrently mitigating macronutrients, heavy metals, emerging pollutants, and pathogenic loads. Backed by their pronounced physiological tolerance to severe environmental toxins, microalgae-based technologies have emerged as a highly promising strategy for the sustainable treatment of municipal, industrial, agro-industrial, and livestock waste streams [55].

In biological wastewater treatment, deploying diverse polyculture consortia—as opposed to monocultures—harnesses ecological niche complementarity and functional redundancy. This multi-species approach enhances community stability, broadens the range of treatable contaminants, and ensures process resilience against fluctuating environmental conditions and toxic shocks [60]. Utilizing mixed-effects modeling, Zhou et al. evaluated the relationship between microalgal alpha-diversity and nitrogen remediation kinetics to elucidate the underlying biological mechanisms governing water quality improvement. Their findings demonstrated a positive correlation between taxonomic diversity and nutrient exhaustion, wherein escalating microalgal diversity significantly enhanced the net nitrogen removal efficiency of the phototrophic community [61].

• Robust metabolic adaptability

Microalgae exhibit profound physiological resilience to fluctuating environmental parameters mediated by phenotypic plasticity, genomic diversity, and symbiotic consortia. Consequently, these organisms serve as critical model systems for elucidating stress-tolerance mechanisms and optimizing ecological interventions in aquaculture, conservation biology, and environmental monitoring. Due to this robust metabolic versatility, strategic strain selection, bioprocess optimization, and genetic engineering represent the core technological pillars required to achieve techno-economic viability in microalgae-mediated wastewater bioremediation [62,63]. Praveen et al. evaluated the efficacy of two acid-tolerant, extremophilic microalgae strains—*Desmodesmus* sp. MAS1 and *Heterochlorella* sp. MAS3—to adapt to the highly alkaline conditions characteristic of winery wastewater. Under mixotrophic cultivation, both strains demonstrated high robustness, achieving rapid specific growth rates alongside significant chlorophyll accumulation. Furthermore, the strains exhibited substantial nutrient remediation capabilities, removing 70–80% of total carbon and 80–90% of both nitrogen and phosphate from the effluent [64]. Investigations by Zhou et al. elucidated the metabolic reprogramming of *Chlorella sorokiniana* subjected to organophosphate ester stress within a synthetic wastewater matrix. The presence of these contaminants induced a functional shift from heterotrophic to autotrophic carbon assimilation, a phenomenon driven by the inhibition of sodium acetate (NaAc) uptake alongside upregulated CO_2_ fixation. Although the microalgae experienced transient growth inhibition initially, complete biomass recovery was achieved by day four, underscoring the profound metabolic plasticity of the strain [65].

#### 2.2.2. Metabolic Flexibility of Microalgae

**Figure 2:**
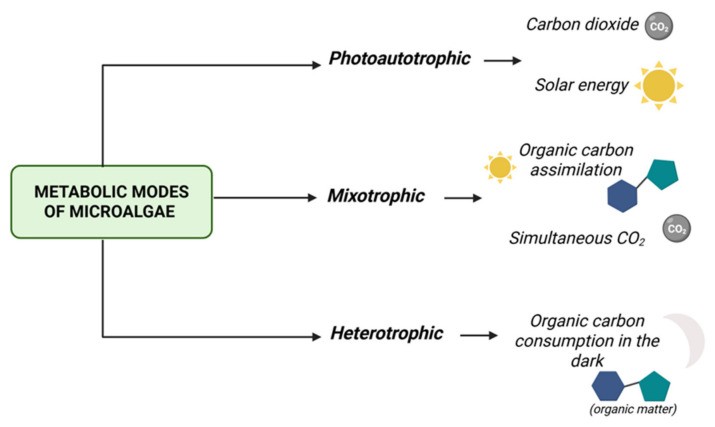
Schematic representation of the primary nutritional regimes in microalgae (created in BioRender, Udrescu M. 2026).

Microalgae execute rapid metabolic reprogramming to shift dynamically among photoautotrophic, mixotrophic, and heterotrophic modes to optimize energy yields and carbon assimilation kinetics (Figure 2).

Significant discrepancies in nutrient reclamation performance across distinct microalgal strains are largely governed by their metabolic plasticity. Specifically, a strain’s capacity to dynamically reconfigure its cellular machinery and transition among photoautotrophic, heterotrophic, and mixotrophic regimes determines its efficacy in processing complex waste streams [13,66] strict photoautotrophs are fundamentally constrained by light attenuation and photon penetration depth within turbid or highly colored effluents—such as cheese whey or industrial wastewater—metabolically flexible strains circumvent these optical limitations by initiating heterotrophic respiration or mixotrophic assimilation pathways [67]. This metabolic versatility enables targeted shifts in nutrient uptake kinetics. Under mixotrophic conditions, the parallel activation of the Calvin cycle and the tricarboxylic acid (TCA) cycle drastically accelerates biomass accumulation rates, resulting in a corresponding stoichiometric increase in the mass-balance removal of nitrogen, phosphorus, and chemical oxygen demand [68]. Consequently, the wide variance in environmental remediation capacities reported in the literature is a direct consequence of strain-specific differences in enzyme upregulation and organic transporter activity during trophic phase-shifts [69].

#### 2.2.3. Nutrient Assimilation, Biosorption, and Microbial Symbiosis

As noted above, microalgae-based remediation systems primarily function by extracting nutrients from wastewater and converting them into valuable algal biomass. This process is intrinsically linked to how microalgae absorb and metabolize carbon, nitrogen, and phosphorus within the effluent. Because toxic contaminants like heavy metals directly threaten biomass yield and subsequent downstream applications, a comprehensive understanding of these cellular interactions is paramount. Unlocking the biological pathways governing nutrient uptake and pollutant detoxification provides the theoretical foundation needed to scale up microalgae-based sanitation and biorefinery systems [5].

##### Carbon (C)

Carbon in wastewater is partitioned into organic fractions (glucose and starch) and inorganic fractions (dissolved CO_2_ and bicarbonate HCO_3_^−^). Microalgae are capable of metabolizing both CO_2_ and the bicarbonate anion during photosynthesis; however, they cannot directly access the carbonate ion (CO_3_^2−^), which only becomes dominant under highly alkaline conditions. Due to the limited diffusion rate of gaseous CO_2_ in water compared to air, aquatic microalgae rely heavily on HCO_3_^−^ as their predominant carbon substrate. Eukaryotic strains subsequently fix this internalized carbon into organic molecules via the Calvin cycle, utilizing the energetic yields of the light reactions (ATP and NADPH) to power their definitive carboxylation, reduction, and regeneration phases. Microalgae could develop carbon-concentrating mechanisms (CCM) in response to the low CO_2_ concentrations. Inadequate CO_2_ supply is a major limiting factor in high-density microalgal cultivation systems. Consequently, supplementing the culture with CO_2_ levels exceeding the standard ambient baseline (0.036–0.042%) has been shown to optimize growth kinetics [24,70]. Reflecting the direct correlation between carbon assimilation and growth, a ratio of 1.882 g of CO_2_ per gram of microalgal biomass was utilized to calculate daily volumetric CO_2_ fixation rates.

In response to low ambient CO_2_ concentrations, microalgae deploy a biophysical Carbon Concentrating Mechanism (CCM). Active transport proteins (such as HLA3 and LCIA) translocate HCO_3_^−^ across the plasma membrane and chloroplast envelope. Once inside the chloroplast, the enzyme carbonic anhydrase (CA) rapidly catalyzes the reversible hydration of bicarbonate into neutral CO_2_. This localizes a high concentration of CO_2_ directly around ribulose-1,5-bisphosphate carboxylase/oxygenase (RuBisCO), minimizing competitive photorespiration. Following the Calvin–Benson–Bassham (CBB) Cycle, eukaryotic strains fix this internalized CO_2_ into organic molecules via three distinct phases: carboxylation—RuBisCO catalyzes the fixation of CO_2_ onto ribulose-1,5-bisphosphate (RuBP), generating 3-phosphoglycerate (3-PGA); reduction—utilizing the energetic yields of the photosynthetic light reactions ATP and NADPH, 3-PGA is reduced to glyceraldehyde-3-phosphate (G3P); and regeneration—G3P molecules are rearranged to regenerate RuBP, while surplus carbon is directed toward downstream starch and lipid synthesis pathways [71].

*Chlorella* sp. UKM2 was successfully used for effluent treatment and CO_2_ fixation. Experimental results demonstrate that integrating wastewater treatment with carbon capture yields significantly higher nutrient reduction and enhanced CO_2_ fixation efficiencies compared to individual, standalone operations [72]. Razzak demonstrated that the microalga *Chlorella protothecoides* achieved optimal biomass productivity along with maximum removal efficiencies of 100% for total nitrogen (TN) and 85% for total phosphorus (TP) when cultivated under a 6% CO_2_ aeration strategy at temperatures ranging from 25 °C to 30 °C [73]. A recent study demonstrates that combining WO_3_/α-Fe_2_O_3_/zeolite photocatalysis with immobilized *Chlorella vulgaris* enhances antibiotic degradation, nutrient removal, and carbon sequestration [74]. The promise of Nile River microalgae, particularly *Scenedesmus* sp., for sustainable wastewater treatment in polluted environments is underscored by the findings of Moghazy and Abdalla [75].

##### Organic Load

Aquaculture wastewater contains organic matter from sources like uneaten feed, fish feces, and dead organisms. As microbes decompose this material, they deplete dissolved oxygen (DO). Under anaerobic conditions, this process can generate toxic by-products, such as hydrogen sulfide (H_2_S) and methane (CH_4_). The system’s organic load is typically measured via biochemical oxygen demand (BOD) and chemical oxygen demand (COD). BOD, which tracks the oxygen consumed during microbial breakdown, generally ranges from 5 to 30 mg L^−1^. Meanwhile, COD measures the total oxygen needed to oxidize all organic and inorganic substances, varying from 30 to 200 mg L^−1^ based on the system’s operational intensity [76]. The primary advantage of integrating microalgae into wastewater treatment is their photosynthetic production of O_2_. This generated oxygen is essential for heterotrophic bacteria to effectively biodegrade organic carbonaceous materials. Microalgal–bacterial consortia facilitate wastewater remediation through a highly efficient metabolic symbiosis. Microalgae assimilate dissolved organic nutrients and concurrently generate molecular oxygen (O_2_) via photosynthesis; this localized oxygenation meets the metabolic demands of heterotrophic bacteria, which catalyze the mineralization of complex organic pollutants, thereby driving a concomitant reduction in both chemical and biochemical oxygen demand (BOD) [77,78].

##### Nitrogen and Phosphorus

Microalgae are highly versatile organisms capable of utilizing nitrogen from both inorganic and organic matrices. Inorganic sources include ammonium, nitrates, and nitrites, while organic sources are amino acids, urea, purines, and nucleosides. Among these options, microalgae exhibit a distinct physiological preference for ammonium when it is accessible. Because ammonium is already in a reduced state, its direct metabolic assimilation and incorporation into cellular biomass requires significantly less energy compared to oxidized forms like nitrate or nitrite. Because microalgae preferentially and more energetically assimilate ammonium over oxidized nitrogen (NO_x_), bacterial nitrification in wastewater treatment increases operational costs, complexity, and nutrient competition by forcing the system to rely on expensive denitrification or longer retention times to remove the remaining nitrate. In microalgae, inorganic nitrogen assimilation is fundamentally linked to carbon metabolism, as it relies on carbon skeletons (keto-acids) to incorporate nitrogen into organic molecules [77]. The assimilation of inorganic nitrogen proceeds via a highly coordinated sequential pathway: transmembrane translocation, enzymatic reduction in oxidized species, and ultimate incorporation into amino acids. First, extracellular nitrogen is transported across the plasma membrane. Oxidized species (NO_3_^−^ and NO_2_^−^) are then reduced to ammonium (NH_4_^+^) through two sequential enzymatic steps: nitrate reduction—cytosolic nitrate reductase (NR) utilizes NADH as an electron donor to transfer two electrons, reducing nitrate to nitrite; nitrite reduction—nitrite reductase facilitates a six-electron transfer powered by reduced ferredoxin (Fd), converting nitrite to ammonium. Consequently, all inorganic nitrogen forms must be reduced to intracellular (NH_4_^+^) before metabolic assimilation. In the final step, ammonium is incorporated into amino acids primarily through the GS/GOGAT pathway. Glutamine synthetase (GS) catalyzes the ATP-dependent incorporation of ammonium into glutamate to form glutamine, while glutamate synthase (GOGAT) transfers the amide group of glutamine to 2-oxoglutarate, producing two molecules of glutamate [79]. Among the studies reviewed, the most frequently utilized microalgae species for municipal wastewater treatment were *Chlorella vulgaris*, *Chlorella sorokiniana*, and *Chlorella pyrenoidosa*. These microalgae achieved nitrogen removal efficiencies ranging from 89% to 100%, outperforming blue-green algae, which showed slightly lower removal rates [80,81,82,83,84,85,86].

While conventional models of microalgae nutrition emphasize the assimilation of inorganic macronutrients, specifically nitrogen (N) and phosphorus (P), diverse taxa exhibit profound metabolic plasticity through the utilization of organic nitrogen substrates. A premier model for this mixotrophic capacity is the chlorophyte *Chlamydomonas reinhardtii*, which orchestrates the catabolism of organic nitrogen via the periplasmic enzyme L-amino acid oxidase 1 (LAO1). This enzyme catalyzes the oxidative deamination of extracellular amino acids, yielding ammonium (NH_4_^+^) and corresponding carbon skeletons. The liberated NH_4_^+^ is subsequently translocated across the plasma membrane for cellular nitrogen assimilation, while the concurrent carbon backbones are integrated into central metabolic pathways (such as the tricarboxylic acid (TCA) cycle) to fuel energetic demands. Consequently, this enzymatic machinery functions as a critical nitrogen-scavenging mechanism, conferring a competitive survival and growth advantage in oligotrophic environments characterized by inorganic nitrogen limitation and organic nitrogen abundance [87].

Phosphorus is a vital element in microalgae, serving as a core structural component of phospholipids and nucleotides, a foundational element of the cellular energy currency, and a key participant in countless metabolic pathways (ATP). In wastewater, inorganic phosphorus (P) speciation is pH-dependent, with microalgae preferentially assimilating HPO_4_^2−^ and H_2_PO_4_^−^ via active H^+^ or Na^+^ symporters driven by plasma membrane H^+^-ATPase. Soluble organic P is also a vital bioavailable source, accessed via extracellular and membrane-bound phosphatases that hydrolyze bound phosphate (PO_4_^3−^) groups. Once inside the cell, energy from photosynthesis or respiration drives the endergonic phosphorylation of ADP to ATP, allowing substrate-level phosphate transfer to organic compounds (e.g., glucose-6-phosphate). Under P-replete conditions, microalgae exhibit “luxury uptake,” accumulating phosphorus beyond immediate metabolic demands to store it as acid-insoluble polyphosphate granules [77].

Beyond biological assimilation, phosphorus is removed from wastewater through physicochemical precipitation, extracellular matrix adsorption, and enzymatic cleavage: chemical precipitation—under highly alkaline conditions (high pH), phosphate reacts with ambient calcium and magnesium ions to precipitate out of solution, primarily via the formation of hydroxyapatite; extracellular adsorption (EPS binding)—extracellular polymeric substances (EPS) secreted by both microalgae and bacteria form hydrogen bonds with phosphate ions, facilitating surface adsorption onto the biofilm matrix; organic phosphorus conversion—complex organic phosphorus is converted into bioavailable orthophosphate through hydrolysis catalyzed by bacterial extracellular enzymes, rendering it susceptible to the same precipitation and adsorption mechanisms. Alternatively, organic phosphorus can bind directly to EPS functional groups on the microalgal–bacterial biomass for subsequent downstream metabolic transformation [88].

Recent studies indicate that microalgal phosphorus removal efficiencies span a broad spectrum, highly contingent upon both the specific algal strains utilized and the characteristic composition of the wastewater stream. Investigating domestic streams, Goncu et al. reported a 57.9% reduction in orthophosphate (PO_4_^3−^) [89]. Utilizing a diverse polyculture of *Chlorella* sp., *Scenedesmus* sp., *Navicula* sp., and filamentous strains, phosphorus remediation efficiencies reached up to 70% in synthetic wastewater matrices [90]. Bench-scale experiments utilizing monocultures of *Chlorella vulgaris* and *Spirulina maxima*, alongside mixed culture consortia, demonstrated total phosphorus reduction efficiencies of 95.8% and 90.4% for untreated and autoclaved secondary domestic effluents, respectively [91].

##### Heavy Metals

Microalgal bioremediation has emerged as a compelling strategy for the sequestration of heavy metals, owing to the straightforward scalability of their cultivation and their robust tolerance across broad operational parameters. While microalgae-mediated systems frequently surpass conventional, non-biological physicochemical methods in heavy metal immobilization efficiency, several systemic challenges impede their widespread industrial scale-up. Microalgae-mediated heavy metal remediation operates through a tripartite mechanism comprising passive, metabolic-independent biosorption—wherein cationic heavy metals are rapidly sequestered by anionic functional groups on the cell wall—alongside active, intracellular bioaccumulation across the plasma membrane and enzymatic biotransformation that alters the chemical valence states of toxic ions to yield less hazardous forms (Figure 1) [92].

Investigations utilizing the green model microalga *Chlamydomonas reinhardtii* have successfully elucidated key molecular components governing molybdenum (Mo) homeostasis, encompassing both molybdate transport mechanisms and molybdoenzyme activities [93,94]. These breakthroughs have subsequently served as architectural blueprints for identifying orthologous molecular machinery across diverse model organisms. Comparative assessments of regional microalgae revealed that the isolates *Chlorella vulgaris* RG1-4 and *Tetradesmus obliquus* Ehr33-9 possessed exceptional resilience and biosorptive capacity for copper, achieving maximum removal capacities of 100.00 mg g^−1^ and 89.73 mg g^−1^, respectively. Concurrently, *Lobochlamys segnis* Ehr31-1 emerged as the premier candidate for cadmium remediation, exhibiting a maximum adsorption capacity of 93.17 mg g^−1^. Taken together, these data underscore the efficacy of using indigenous, locally adapted microalgal ecotypes as robust biosorbents for the decontamination of heavy metal-polluted aquatic matrices [95].

##### Algae–Bacteria Symbiosis

Bacteria and microalgae constitute two foundational ecological guilds, each possessing distinct physiological frameworks and specialized functional roles. Upon establishing a mutualistic symbiosis, these domains interface to form a highly efficient micro-ecosystem wherein metabolic dependencies are structurally balanced [96]. In diverse natural aquatic ecosystems and engineered constructed wetlands, microalgae drive primary productivity via photosynthesis, yielding dissolved oxygen and labile carbohydrates. This localized organic and oxic flux is subsequently metabolized by heterotrophic bacteria to fuel biomass maintenance and the decomposition of complex organic matter, concurrently mineralizing and releasing inorganic nutrients that are re-assimilated by microalgae and macrophyte communities (Figure 1) [15]. A substantial body of recent literature demonstrates that synergistic microalgal–bacterial consortia represent highly effective and sustainable platforms for wastewater bioremediation. Extensive literature highlights the superior bioremediation efficiency of these consortia when deployed in aquaculture effluents. For example, indigenous algal–bacterial co-cultures achieved robust pollutant mitigation, yielding removal efficiencies of 82.6% total nitrogen (TN), 93.5% ammonium-nitrogen (NH_4_^+^-N), and 70.6% chemical oxygen demand (COD) in high-strength turtle cultivation discharge. Similarly, in anaerobic sludge liquors, the system stripped 87.1% TN, 91.7% COD, 77.1% NH_4_^+^-N, and 89.0% total phosphorus (TP)—persistently outperforming non-algal control configurations by a margin of 25% to 45% [97,98,99]. Consortia between microalgae and nitrogen-fixing bacteria are particularly relevant in the context of low nitrogen-containing wastewater streams. This mutualistic symbiosis consists of the diazotrophic bacteria providing biologically fixed nitrogen to the algal partner, while microalgae supply oxygen, organic carbon, and photosynthetically derived metabolites supporting bacterial activity, leading to an overall improved biomass productivity, reducing the need for external nitrogen supplementation, and enhancing the overall sustainability of wastewater-based microalgal biorefineries [100].

#### 2.2.4. Primary Parameters Affecting Treatment Performance

The significant variance in pollutant removal kinetics observed for identical microalgal strains across the literature can be fundamentally attributed to three core parameters that dictate systemic performance.

• Light Intensity and Photoperiod

Solar or artificial irradiance serves as the primary energetic driver governing photoautotrophic microalgal proliferation and subsequent wastewater bioremediation efficiency. Optimizing photosynthetic productivity inherently depends on tailored light–dark regimes, which balance the enzymatic dark reactions of carbon fixation with initial photon capture. Furthermore, light intensity fundamentally modulates secondary system parameters—specifically pH, temperature, and dissolved oxygen profiles—all of which exert critical feedback loops on cellular kinetics. Consequently, the distinct impacts of photon flux density (light intensity), photoperiodicity, and spectral distribution (wavelength) have been extensively characterized under controlled laboratory conditions. To assess the impact of irradiance on *Chlorella sorokiniana* cultivated in nitrogen-rich anaerobic digestate, a 30-day experiment was conducted using three light intensities (20, 68, and 162 µmol m^−2^ s^−1^) under a 16:8 h photoperiod. Higher irradiances dramatically accelerated both growth and remediation efficiency. At 162 µmol m^−2^ s^−1^, biomass production was five times greater than at 20 µmol m^−2^ s^−1^. This metabolic spike also expedited wastewater treatment; high-light-driven cultures cleared 94% of ammonium nitrogen and 100% of phosphorus within 7 days. In contrast, low-light cultures achieved only 55% ammonium removal over the full 30 days, while phosphorus was completely eradicated within the first week at both 68 and 162 µmol m^−2^ s^−1^ [101]. The impacts of temperature, photoperiod, and light intensity on the cultivation and nutrient remediation potential of *Desmodesmus* sp. CHX1 in turtle aquaculture wastewater was investigated. The highest nutrient clearing was established at 30 °C with continuous light at 180 μmol photons m^−2^ s^−1^; these parameters yielded removal efficiencies of 86.53% (ammonia), 97.94% (nitrate), 99.57% (nitrite), and 99.15% (total phosphorus). Statistical trends indicated that TP removal was insensitive to temperature fluctuations, whereas increments in both photoperiod and photon flux density strictly governed its removal performance [102].

• Temperature gradients

As a primary physical stressor, temperature fundamentally dictates microalgal metabolic pathways. It strongly regulates cellular lipid profiles, nutrient assimilation kinetics, carbon fixation, and overall proliferation rates. Thermal stress alters cytoplasmic viscosity, which subsequently impairs the efficiency of cellular carbon and nitrogen utilization. Furthermore, temperature acts as a critical modulating factor in photoinhibition (light-induced damage to the photosynthetic apparatus), directly impacting biomass accumulation. Typically, the microalgae growth rate exhibits a thermodynamic relationship: accelerating alongside rising temperatures up to a species-specific optimum, beyond which any further thermal increase induces a sharp decline in growth [103].

A study investigates the potential of phototrophic microalgae, specifically *Chlorella protothecoides*, for biological wastewater treatment, with a focus on the effects of air temperature and CO_2_ concentration on nutrient removal from tertiary municipal wastewater. The impacts of temperature on microalgae *Chlorella protothecoides* growth and nutrient reduction kinetics were evaluated. Maximum biomass accumulation was observed at 25 °C, while elevated temperatures (30 °C, 40 °C, and 45 °C) inhibited growth performance. However, maximum total nitrogen (TN) and total phosphorus (TP) assimilation occurred within a slightly warmer range of 25–30 °C, culminating in 100% TN removal and 85% TP removal at a stabilized cultivation temperature of 30 °C. *Chlorella vulgaris* was grown in municipal wastewater for 15 days to compare the remediation and growth performance of three temperature profiles: constant 4 °C, constant 35 °C, and a fluctuating 35 °C day: 4 °C night cycle. The diurnal alternating regime outperformed both continuous setups, maximizing final biomass (1.62 g L^−1^) and daily productivity (99.21 mg L^−1^ day^−1^). This temperature profile also drove the most effective wastewater purification, eliminating 83.0% of COD, 96.5% of TN, 97.8% of NH_3_-N, and 99.2% of TP [104].

• pH

Environmental pH strongly regulates microalgal development by controlling the activation of key proteins and enzymes. While neutrality is globally preferred, specific microalgae display remarkable physiological plasticity, tolerating extreme acidic (pH < 5) or alkaline (pH > 9) brackets. This relationship is bidirectional: microalgal growth actively modifies the surrounding medium’s pH through a complex proton imbalance. Specifically, the consumption or generation of cellular alkalinity is heavily dictated by the choice of nitrogen substrate and its metabolic processing, which alters the culture’s pH profile to a greater extent than CO_2_ assimilation alone [105]. The natural increase in pH, nutrient assimilation kinetics, and biomass yields were characterized within a cyclical microalgal re-cultivation system engineered for consortium adaptation to secondary WWTP effluent. A progressive elevation of media pH to values of 9.7, 9.8, and 9.9 was observed, which likely facilitated the complete removal of fecal bacteria below detectable values. Furthermore, the deployment of this adapted consortium across batch cycles 1 to 3 yielded an increase in biomass productivity ranging from 66% to 167%. This accelerated metabolic activity ultimately resulted in significant reductions in total alkalinity (13.1–50.3%), NO_3_-N (30.9 to 53.6%), PO_4_^3–^-P (90.4 to 93.0%), and electrical conductivity (EC) (8.5 to 18.1%) [106].

Three pH control strategies were tested on a mixed microalgal consortium cultured in diluted anaerobic digestate: uncontrolled (R_UC_), stabilized at pH 7–8 (R_7–8_), and capped below pH 8 (R_8_). In RUC reactors, an early biogenic pH spike ≤ 8.6 led to substantial non-biological NH_4_^+^-N loss via stripping. The implementation of strict pH control heavily dictated system performance: R_7–8_ maximized microalgal growth rates and biological NH_4_^+^-N capture, while R < 8 achieved superior biomass settleability and successfully suppressed bacterial growth relative to the microalgae [105].

## 3. Added Value Bioproducts from Wastewater-Grown Microalgae

### 3.1. Bioenergy and Biofuels

Microalgae present several attractive characteristics for use in energy recovery and fuel production, such as rapid growth as a result of their high CO_2_ fixation, low lignin content, and several valorization pathways due to their high content of lipids and carbohydrates [107]. Additionally, provided sufficient light exposure and basic mineral nutrients, microalgae can grow in a wide variety of aqueous media, including various sources of wastewater. Recent research has been focused on expanding the valorization of microalgae in a dual integrated approach, wastewater treatment and energy recovery in the form of biofuels [108,109]. Other strategies involve identifying and testing consortia either with multiple strains of microalgae or microalgae and other microorganisms such as yeasts, fungi, and bacteria. The aim being to take advantage of the synergistic interactions that complement each other’s deficiencies regarding specific toxicity and temperature variation vulnerability [110,111,112]. The addition of fungal pellets of *Aspergillus niger* has been shown to have dual advantages for algal harvest and contributing to nutrient removal in cassava biogas effluent wastewater, specifically phosphorus and COD [113].

#### 3.1.1. Hydrothermal Conversion Products

One potential research direction for energy recovery in the form of biofuels obtained from wastewater-grown microalgae involves producing sustainable aviation fuel (SAF) and SAF precursors. Investigations focus on the hydrothermal liquefaction (HTL) of wastewater-grown microalgae in order to obtain bio-oil, which exhibits similar chemical compositions to fossil origin petroleum. In the study of Marangon et al. [114], the one-step HTL of *Chlorophyceae*–*Bacillariophyceae* consortium used in the treatment of wastewater resulting from the meat-processing industry, resulted in a maximum oil yield of 23.07% after 30 min at 320 °C, using 10% NiMo/Al_2_O_3_ catalyst, while the highest heating value, 41.77 MJ kg^−1^, required 90 additional minutes to be achieved. *Chlorella sorokiniana*, isolated and grown in municipal wastewater, not only exhibited an enhanced lipid production of 31% SAF-equivalent components but also achieved a 10% higher biomass productivity in wastewater over commercially available cultivation medium AF-6. Multiple cultivation scenarios investigated, highlighted the aeration strategy and drying step as crucial elements towards reducing negative environmental impact [20]. HTL processing of municipal wastewater-grown microalgae polyculture, resulting from a fixed-film bioreactor at the treatment facility in Iowa City, IA, USA, led to mass and carbon recoveries of 26% and 44%, respectively. However, techno-economic analysis highlighted the requirement for improvements to be made to achieve economic competitiveness with fossil fuels [115]. Some of the limitations towards industrial scale-up remain high capital costs, severe reactor corrosion, as well as the technical difficulties in processing high-ash biomass, which could remain a bottleneck [116]. Alternative pathways for the extraction of lipids from microalgae for SFA production involve the use of ionic liquids due to their affinity for polar lipid fractions and glycolipids [117]. Co-hydrothermal liquefaction of microalgae and dairy sludge at a ratio of 3 to 1 resulted in bio-oil yield similar to the one obtained in the scenario of using microalgae as mono-feedstock, 32.94% to 33.50%. However, the oil obtained from the co-HTL process exhibited better characteristics, such as lower O/C and higher H/C ratios, improved carbon recovery, better calorific value, with a heating value of 38.24 MJ kg^−1^, and decreased levels of N-heterocyclic and aromatic compounds [118]. Municipal wastewater-grown consortia containing *Microcystis aeruginosa*, *Scenedesmus quadricauda*, and *Pediastrum gracillimum*, alongside cyanobacteria, were subjected to HTL for biofuel production. Optimal parameters were identified to be 250–300 °C, for 30 min at a biomass-to-water ratio of 1:10, producing a biofuel rich in aliphatic hydrocarbons, achieving an energy recovery of 35.79%. Product yield represented 16.86% biofuel, with a heating value of 42.93 MJ kg^−1^, consisting of desirable fractions such as 11.37% gasoline, 29.41% kerosene, 9.71% diesel, but challenging factors remain scalability, feedstock variability, and the necessity to use all byproducts to improve sustainability [119]. Economic analysis of the feasibility of bio-oil from wastewater-grown microalgae via hydrothermal liquefaction highlights that costs remain high, especially cultivation costs, which accounted for half to two-thirds of the overall expenses, leading to the products being commercially non-competitive [120]. An alternative hydrothermal processing pathway, hydrothermal carbonization (HTC) of wastewater-grown microalgae, can be used to obtain hydrochar. Applying an acid pretreatment has been shown to improve characteristics of hydrochar as well as enhance energy and carbon recovery; however, the drawback of the elevated nitrogen, sulfur, and heavy metals levels requires additional adjustments [121].

#### 3.1.2. Biodiesel

Microalgae cultivation using wastewater or modified wastewater as growth medium is potentially a more sustainable approach for biodiesel production, being more cost-effective while simultaneously recovering nutrients from wastewater [122]. In order to facilitate the integration of microalgae-derived biodiesel on the fuel market, extensive research must be conducted on optimizing the cultivation of microalgae strains with high lipid content and rapid growth, under adverse conditions, at a low cost. Regarding the transesterification process, the biorefinery approach consists of developing alternative and efficient catalysts, which can be reused with minimal changes in yield, and reduced generated waste [123,124]. *Marvania coccoides*, cultivated in nutrient-optimized wastewater, has been investigated for biodiesel production, achieving a biomass productivity of 937.5 ± 17.68 mg L^−1^ and a lipid content of 19.8 ± 0.99%. Ex situ and in situ transesterification processes were carried out with immobilized lipase, the highest fatty acid methyl esters (FAME) yields being 91.95 ± 0.32% and 72.54 ± 0.84%, respectively. Ultrasonic process intensification further increased in situ yield to 90.65 ± 0.77%. The simultaneous wastewater treatment and transesterification using a reusable immobilized enzyme, with a yield of 64.2 ± 0.84% in the fourth reaction cycle, could prove a sustainable approach but still requires investigation regarding scale-up potential and feasibility [125]. An attempt to upscale a continuous photobioreactor to pilot scale for microalgal biomass production from wastewater resources and conversion into biodiesel identified positive net energy ratios (NER = 4.95–8.38) for biomass production. However, the high energy requirements of biomass processing and biodiesel synthesis, estimated to be up to 90% of the total energy demand, led to negative values for biodiesel production (0.14–0.23) [126]. Two green microalgae species, *Scenedesmus obliquus* and *Chlorella vulgaris*, grown in dairy wastewater treated by activated sludge systems, yielded maximum biomass yields of 2.55 g L^−1^ and 3.10 g L^−1^, respectively, in batch-operated photobioreactors. *Scenedesmus obliquus* also accumulated the highest values for lipids and carbohydrates, 21% and 39%, respectively. Mass balance analysis highlighted the great potential for biofuel production, of 284 g biofuel per kg of biomass if both valorization pathways are explored [127]. An alternative valorization pathway for dairy-industry wastewater is based on using stormwater for diluting dairy wastewater and providing growth medium for a yeast–microalgae consortium consisting of *Saccharomyces cerevisiae* and *Scenedesmus abundans* for biomass and biofuel production. Alongside a significant reduction in nutrient content from the dairy wastewater, 41.7% of total nitrogen, 60.9% of total phosphorus, 83% of COD, and 90% of BOD, an increase in biomass accumulation of approximatively 58% and lipid accumulation of 31% over the control samples was also reported [128]. An oscillating grid device coupled with agricultural phytohormones was used to enhance the biomass and lipid accumulation in *Scenedesmus quadricauda*. Optimal operating conditions consisted of moderate turbulence and added phytohormones, which allowed for an increase in biomass concentration by 24.78% and lipid yield by 70% to be achieved in comparison to stationary samples [129].

#### 3.1.3. Digestion and Co-Digestion of Wastewater-Grown Microalgae

More widespread uses of microalgae in biogas installations revolve around the use of microalgae for nutrient recovery from diluted anaerobic digestion effluents. Green algae, such as *Chlorella sorokiniana* and *Chlorella vulgaris,* showed good ammonium removal efficiencies when used in 10–20% anaerobic digestion effluent, while for strains from the *Scenedesmaceae* family, a more diluted effluent, up to 10%, was necessary for efficient microalgae growth [130]. Similarly, for *Desmodesmus* sp., it was shown that modifying the anaerobic digestion effluent by adding supplementary nutrients, such as magnesium, iron, and phosphorus, not only effectively doubled biomass production over non-modified wastewater, 0.78 g L^−1^ to 0.35 g L^−1^, but it also performed better than standard microalgae cultivation medium BG11, with a 44% increase in biomass production. Additionally, the modified anaerobic digestion effluent yielded higher contents of valuable products, 23.98% aliphatic hydrocarbons and 42.33% fatty acids, and reduced content in potentially toxic nitrogen compounds [131]. Significantly increased attention is given to the potential of microalgae to also be used as feedstock for digestion processes, and besides their biogas potential, to also look into the environmental impact of these processes through life cycle assessment tools and models [132]. Microalgal biomass consortium obtained from tertiary wastewater treatment of a pilot-scale facility has been investigated through the lens of biogas potential. Energy balance calculations showed that implementing an anaerobic digestion step could potentially reduce the energy of the plant by 20% and create an energy surplus [133]. The feasibility of microalgae-derived biogas can be improved by applying unconventional pretreatment options, such as ultrasound, with the caveat that a techno-economic analysis is necessary to ensure a balance between biogas production and energy demand. Experiments carried out on *Chlorella vulgaris* cultivated in animal wastewater showed that ultrasonic pretreatment facilitated biogas production by disrupting microalgae cells. Although the highest biogas yields achieved were in the 680–690 mL g^−1^ COD range, depending on the operating conditions of the ultrasonic pretreatment, only shorter treatments were able to achieve positive energy gains, at the expense of biogas productivity, which was 541 mL g^−1^ [134]. Microalgae have also been investigated in co-digestion scenarios. Javed et al. proposed the co-digestion of *Chlorella vulgaris* with wastewater activated sludge, noticing that methanogenic activity was favored or suppressed depending on whether or not the feedstocks and nutrients were pre-mixed or not. By using separate streams, hydrogen production was enhanced instead of the methanogenic processes, leading to minimal CO_2_ production [135]. *Coelastrella striolata* var. *multistriata* strain 047 was identified as a potential option due to its being an effective bioremediation agent with a composition that favors energy recovery in the form of biofuels. Although several scenarios were reported, the only feasible directions of valorization were those that recovered more than one type of bioproduct, respectively, biocrude, biogas, and fertilizer. Identifying unused energy sources on-site could also improve sustainability [136]. Natural fermentation of microalgae–bacteria consortia from domestic wastewater treatment ponds, containing the microalgae *Tetradesmus obliquus*, *Chlorella vulgaris*, *Scenedesmus acunae*, and *Nitzschia*, has been investigated as a potential source of biohydrogen. Although thermal pretreatment enhanced biohydrogen yield, it also extended the lag phase and decreased productivity, which for untreated biomass reached after 5 days of fermentation reached 1.25 ± 0.08 mmol H_2_ h^−1^ [137]. An approach more in line with the concepts of circular bioeconomy would be to incorporate in a biorefinery the simultaneous recovery of high-value-added compounds as well as bioenergy. Urban wastewater-grown microalgae have been shown to have comparable methane yields between raw and carotenoid-extracted biomass, 159 vs. 136 NmL CH_4_ g^−1^ volatile solids. The efficiency of methane production can also be improved by co-digestion of the spent biomass with other substrates, such as sludge [138].

#### 3.1.4. Bioethanol

When viewing microalgal biomass through the lens of bioethanol production, one of the main advantages over other feedstocks is represented by the considerably high percentage of fermentable hexoses. The lack of lignin avoids problems such as biomass recalcitrance, the necessity for harsh and costly pretreatment steps, and the generation of inhibitors throughout the fermentation process [139]. The dual role of wastewater treatment and bioethanol production played by microalgae could be alternatively fulfilled by cyanobacteria, such as *Dolichospermum spiroides*, but concerns remain regarding the toxicity to human health and aquatic ecosystems, and scale-up attempts, even at a 10 L pilot-scale trial, showed performance issues over laboratory scale, requiring additional research [140].

Microalgae *Chlorella vulgaris* PSP-E, isolated from freshwater in southern Taiwan, was grown in modified BG-11 medium, achieving 60% carbohydrates accumulation using 25% swine wastewater and 2% CO_2_–air mixture. After acid hydrolysis, the hydrolysate composed of glucose and galactose was subjected to fermentation by commercially available *Saccharomyces cerevisiae*, yielding 0.22 ± 0.01 g ethanol per g biomass within 8 h [139]. *Marvania coccoides*, a strain closely related to the *Chlorella genus* isolated from a freshwater pond in India, was tested in both laboratory and open-pond scenarios for bioethanol production. The culture medium used was BG11 modified with 2% anaerobic digestion wastewater, yielding 73.50 ± 0.07 mg L^−1^ d^−1^ biomass at lab scale, respectively, 84.58 ± 1.77 mg L^−1^ d^−1^ in a 200 L open pond. Alongside the high nutrient reduction rates (97.16 ± 0.47% ammonia, 80.20 ± 0.57% nitrate, 73.80 ± 0.95% phosphate, and 75.28 ± 3.15% COD), the harvested biomass cultivated in the scaled-up scenario contained 47.57 ± 1.04% carbohydrates, yielding 65.80 ± 0.25 mg g^−1^ bioethanol within 24 h [141].

Microwave has been shown to enhance fermentable sugars release during acid pretreatment of *Chlorococcum* sp. cultivated in mixed dairy and paper-pulp wastewater, with a maximum yield of 15.67 g L. Further simultaneous saccharification and fermentation of pretreated microalgal biomass allowed for a maximum bioethanol production of 7.85 g L^−1^ to be achieved, corresponding to a bioethanol productivity of 0.98 g L^−1^ h^−1^ [142]. A novel approach to microalgae fermentation to bioethanol involves more unconventional reagents for the chemical pretreatment of the microalgal biomass and a more thorough evaluation of the downstream performance of microalgae-derived bioethanol. One proposed pathway consists of pretreatment of microalgae *Chlorella vulgaris* with thionyl chloride and hydrolysis by *Trichoderma viride* prior to fermentation by *Saccharomyces cerevisiae*, yielding 18.2 g L^−1^ ethanol, which was further investigated in bioethanol-gasoline blends, highlighting the lower hydrocarbons, carbon monoxide, and carbon dioxide emissions over pure gasoline [143]. An alternative to bioethanol, biobutanol is considered the superior fuel, having a higher energy density and being more compatible with gasoline. This allows for blends of higher ratios to be used before requiring engine modifications while also being less corrosive. Species such as *Tetraselmis subcordiformis*, *Chlorella vulgaris*, *Chlamydomonas reinhardtii*, and *Scenedesmus obliquus* have been identified as suitable substrates for biobutanol production due to their high starch and convertible sugar contents [144]. *Hindakia tetrachotoma*, grown in Bold Basal Medium mixed with municipal wastewater and added polypropylene and polyethylene, has been investigated in a dual valorization approach, which could potentially offset some of the costs associated with either biobutanol or carotenoids production. The highest biobutanol productivity was achieved under stress conditions by adding 75 mg L^−1^ of PP and PE, at 0.040 ± 0.001 g g^−1^ biomass, significantly higher than the control sample without added microplastics, at 0.024 ± 0.001 g g^−1^ biomass. Additionally, with increasing amounts of microplastics, a directly proportional increase in carotenoid concentration was observed in response to the external stress factors [145]. Large-scale production of bioethanol and biobutanol derived from wastewater-grown microalgae is limited by technical, biological, economic, and environmental challenges, which collectively decrease feasibility. The structural complexity and heterogeneity of cell walls, one of the main technical barriers, require intensive mechanical, chemical, or enzymatic pretreatments, which are limited by low conversion efficiency, high reagent or energy costs, and the formation of inhibitory by-products. In the specific case of biobutanol, additional limitations that further hinder process standardization and commercial feasibility include low solvent yields, product toxicity to fermenting microorganisms, and the variability of wastewater-grown biomass composition [146].

### 3.2. Fertilizers and Biostimulants

Microalgae are well established as potential biostimulants and a source of compounds that can promote growth in plants, such as polysaccharides, phytohormones, and proteins [147]. For a substance to be considered a biostimulant, it has to boost nutrient use and availability, improve plant quality, or enhance tolerance to stress factors. Research on wastewater-cultivated microalgae for biostimulant production is still limited, but promising due to considerations regarding economic and environmental sustainability [148,149,150]. The capacity for storage inside microalgal cells of nitrogen and phosphorus by removing nutrients from wastewater is a determining factor towards the effectiveness of microalgae to fulfill the dual role of bioremediation and biofertilizer production. Although microalgae species *Chlorella*, *Spirulina*, and *Scenedesmus* have shown high removal rates for municipal, poultry, and dairy wastewater, differences in composition, such as low phosphorus loading in poultry wastewater and or excess organic matter in dairy wastewater, highlight the need for different strategies to optimize environmental impact, tailored to specific combinations of microalgal species and wastewater origin [151,152].

Urban wastewater-grown microalgae consortium containing mainly *Scenedesmus* sp. was shown to accumulate high concentrations of phytohormones, such as auxins, cytokinins, and gibberellins. By using distilled water extracts from this consortium, a positive effect on the growth of lettuce in well-watered conditions was shown [148]. Similarly, microalgae biomass harvested from a pond treating food waste digestate, also consisting mainly of *Scenedesmus* sp., was used as biofertilizer for *hybrid Brachiaria* cv. *Sabiá* is a forage plant commonly used in Brazil as animal pasture. Raw microalgal biomass, as well as anaerobically digested biomass and co-digested with food waste, were compared to inorganic fertilizers. Dry matter productivity was similar between microalgae-based and inorganic fertilizations, with the leaf phosphorus content being 21% higher when fresh microalgal biomass was used over the control. For digested and co-digested microalgae treatments, soil analysis showed an increase of 8–12% organic matter, 10% nitrogen, and 6–20% phosphorus [153]. A multi-pronged approach can potentially increase the sustainability and profitability of biostimulant production by further downstream valorization of spent microalgal biomass. *Scenedesmus* sp. biomass was grown in a demonstrative raceway pond using diluted recycled nutrient media from inorganic fertilizers and subjected to ultrasonic pretreatment and enzymatic hydrolysis for biostimulants production. Bioassays were carried out on watercress, mungbeans, and wheat, confirming a beneficial effect of the microalgae-derived extract, similar to gibberellins, auxins, and cytokinins. Additional processing of the spent biomass has shown that the extraction step improved methane yield by 20% and increased the kinetics of the anaerobic digestion process by 10% over the raw microalgal biomass, attributed to the disruption of the microalgal cell wall [154]. Laboratory-scale as well as a 950 L closed tubular reactor were investigated for the cultivation of *Desmodesmus* sp. in waste stream from greenhouse hydroponic drainage with supplemented nutrients, for their potential on-site use. Microalgal biomass was recovered by flocculation with iron chloride, and biostimulant production was carried out in enzymatic processes, achieving a maximum of 74% reduction in nitrate levels and complete retention of phosphorus, potassium, and calcium [155].

### 3.3. High-Value-Added Products

Microalgae cultivation for simultaneous pollutant removal from wastewater and biomass valorization for high-value bioproducts and biopolymers represents a promising direction in line with the concepts of a circular bioeconomy, but integration of wastewater treatment with recovery of high-value products is still limited [156,157].

Carotenoid biosynthesis in microalgae occurs via the methylerythritol phosphate pathway, initiated by glycolytic metabolites, glyceraldehyde-3-phosphate and pyruvate, which are jointly converted into universal C5 precursors, isopentenyl pyrophosphate and dimethylallyl pyrophosphate. Environmental stress factors, such as fluctuations in temperature or light source, nutrient deficiency, and the use of exogenous hormones, can activate the expression of specific genes, such as key carotenogenic genes, improving their tolerance to environmental stress and promoting carotenoid accumulation [158,159]. Microalgae such as *Chlorella* sp., *H. pluvialis*, and *Scenedesmus* sp. have been shown to accumulate carotenoids when used for the bioremediation of wastewater rich in starch from processing industries of corn, potato, and cassava [160]. Canteen wastewater has been successfully used for *Spirulina platensis* growth, achieving a three-fold increase in both biomass productivity, at 0.071 g L^−1^ day^−1^, and carotenoid accumulation, at 21.81 mg g^−1^ dry weight, over control samples. Additionally, wastewater remediation was highly efficient, with 92.12% nitrate and 90.05% phosphate being removed [161]. A microalgae consortium consisting of *Chlorella vulgaris* and *Tetradesmus* was shown to accumulate chlorophylls, carotenoids, and biomass when used in the wastewater treatment of paint booth effluent from the wooden furniture varnishing process, mixed with domestic sewage. These results have been linked with the presence of volatile organic compounds in paint booth effluent, which offset the carbon deficiency of domestic sewage [162]. A direction that could potentially allow for simultaneous municipal wastewater treatment and carotenoid accumulation while also reducing harvesting costs was tested at both laboratory and outdoors in a 30 L conical reactor, using a microalgal consortium consisting mainly of *Chlorella* sp., *Scenedesmus* sp., and cyanobacterium *Leptolyngbya*. The proposed harvesting method involves the recultivation of settled microalgae to enhance flocculation. The obtained biomass had added antioxidant value due to the presence of 451 μg carotenoids g^−1^ dry weight, which could potentially be used for agricultural systems [163].

*Spirulina* cultivated in an open pond with nutrient-adjusted domestic wastewater was investigated for an integrated biopolymer-biochar production approach, reaching a maximum yield of 0.83 g L^−1^ after 12 days. The biomass was subjected to methanol-water extraction for phycocyanin recovery, yielding 22.5 mg L^−1^. Residual algal biomass was further subjected to hydrolysis with sodium hypochlorite for polyhydroxyalkanoates (PHAs) production, which were used in a bioplastic formulation that showed good biodegradability. The spent biomass was used for biochar production, yielding 31% biochar by torrefaction [164]. Alternatively, microalgae have also been utilized in a membrane-separation dual-chamber reactor for bacterial multiculture PHA production, for supplying oxygen and reducing CO_2_ emissions, therefore reducing some of the associated costs by lowering specific energy use [165]. *Porphyridium cruentum* was studied in artificial seawater-based Walne medium with added glycerol and tofu wastewater as carbon sources, for dual sulfated polysaccharide biopolymer production and wastewater remediation. For optimum conditions identified, biomass productivity reached 14.13 g L^−1^, while intracellular cell-bound polysaccharides and extracellular polysaccharides were 0.67 g L^−1^ and 0.61 g L^−1^, respectively [166]. *Arthrospira platensis* and *Parachlorella kessleri* have been proposed as potential candidates for biopolymers production after cultivation in secondary-treated wastewater due to their effectiveness in nutrient recovery and adaptability to various environmental conditions [167]. A different pathway for waste-grown microalgal biomass in the field of biopolymer production consists of partial substitution of polylactic acid with microalgae biomass from a wastewater plant treating sanitary sewage. Lower concentrations of microalgae (10–20%) enhanced mechanical performance, reducing surface defects and leading to a more cohesive matrix, while formulations with higher amounts of microalgae improved biodegradability. The polylactic-microalgal biomass composite achieved a degradation rate of 89% after 120 days; however, the odor specific to waste-grown microalgae significantly limits applicability to the agricultural sector [168].

## 4. Techno-Economic Analysis and Life Cycle Assessment of Bioproducts Obtained from Microalgae-Based Wastewater Treatment

Following the review of the major bioproduct categories derived from wastewater-grown microalgae (biofuels, biofertilizers, biopolymers, and high-value compounds), it is essential to evaluate whether these products can be produced in an economically viable and environmentally sustainable manner. Techno-economic analysis (TEA) and life cycle assessment (LCA) provide the primary frameworks for such evaluation.

### 4.1. Methodological Foundations of TEA and LCA for Wastewater Derived Microalgal Bioproducts

**Figure 3:**
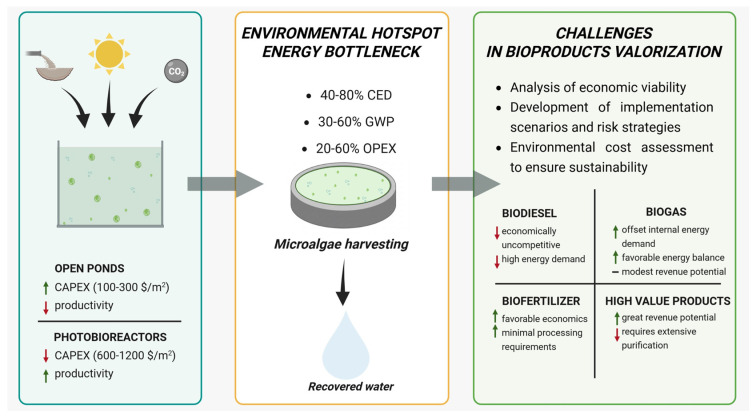
Flowchart for microalgae-based wastewater treatment up to products, highlighting the main bottlenecks and challenges of the process (created in BioRender, Udrescu M. 2026).

Techno-economic analysis (TEA) and life cycle assessment (LCA) have become indispensable tools for evaluating the feasibility of bioproducts derived from microalgae cultivated in wastewater [169]. Over the past decade, the research focus has shifted from assessing microalgae primarily as wastewater treatment agents to evaluating their potential as feedstocks for biofuels, biofertilizers, biogas, animal feed, and high-value bioactive compounds [170]. This transition reflects a broader recognition that the long-term sustainability of microalgae-based systems depends not only on nutrient removal efficiency but also on the economic and environmental performance of the resulting bioproducts [171]. As a result, TEA and LCA are increasingly applied in an integrated manner to capture the complex interactions between cultivation conditions, biomass composition, processing requirements, and product valorization pathways [169,170]. Figure 3 shows an overview of TEA and LCA of bioproducts derived from wastewater-grown microalgae, as well as challenges, advantages, and disadvantages of the more common valorization pathways.

#### 4.1.1. TEA: Cost Structure, Product Yields, and Economic Feasibility

From a TEA perspective, the economic feasibility of wastewater-derived microalgal bioproducts is strongly influenced by both upstream and downstream factors. Upstream, cultivation costs vary widely depending on reactor configuration and wastewater characteristics. Open raceway ponds typically exhibit capital expenditures (CAPEX) of 100–300 USD m^−2^, whereas tubular or flat-panel photobioreactors may exceed 600–1200 USD m^−2^ due to higher material and operational requirements [172,173]. These differences translate into substantial variation in biomass production costs, which range from 0.5 to 4.0 USD kg^−1^ depending on productivity, climatic conditions, and nutrient availability [174]. Wastewater integration can reduce nutrient-related operational expenditures (OPEX) by 30–70%, as demonstrated in municipal and agro-industrial wastewater systems [175]. However, the economic benefits of nutrient substitution are often offset by increased variability in biomass composition, which directly affects product yields [2].

The biochemical composition of microalgae biomass (proteins, lipids, and carbohydrates) dictates downstream processing efficiency and costs, yet it remains highly sensitive to seasonal and daily fluctuations in wastewater composition. This variability influences biomass quality through three primary mechanisms: nutrient (N:P) ratios, where nitrogen limitation shifts metabolism away from protein synthesis toward lipid or carbohydrate accumulation; shifting organic loads (COD/BOD), which induce mixotrophic or heterotrophic growth modes that alter carbohydrate and lipid profiles; and the presence of heavy metals or micropollutants, which induce physiological stress, increase intracellular ash content, and reduce overall biomass purity [2]. For instance, lipid content in wastewater-grown microalgae typically ranges from 10 to 35%, lower and more variable than the 20–50% commonly reported for synthetic media [176]. This variability contributes to the wide range of reported biodiesel production costs, from 6.8 USD kg^−1^ to over 11 USD kg^−1^ [177,178]. Similarly, methane yields during anaerobic digestion vary between 200 and 350 mL CH_4_ g^−1^ volatile solids, depending on wastewater type and pretreatment [179]. For high-value compounds such as carotenoids or phycobiliproteins, wastewater-derived biomass often requires additional purification steps to remove contaminants, increasing processing costs by 20–60% compared to biomass grown on sterile media. These examples illustrate the central methodological challenge in TEA: wastewater improves nutrient economics but introduces uncertainties that propagate through the entire value chain [175].

#### 4.1.2. LCA: System Boundaries, Functional Units and Environmental Indicators

LCA provides a complementary perspective by quantifying the environmental impacts associated with each stage of bioproduct generation. System boundaries vary depending on the product: cradle-to-gate for biodiesel and biofertilizers, cradle-to-product for pigments and nutraceuticals, and cradle-to-grave for biogas and energy applications [180]. Functional units also differ, ranging from 1 kg of biodiesel or biofertilizer to 1 MJ of biogas or 1 g of purified pigment. Across studies, harvesting and drying consistently emerge as environmental hotspots, contributing 40–80% of cumulative energy demand (CED) and 30–60% of global warming potential (GWP) [181,182]. In contrast, cultivation in wastewater often yields environmental credits due to avoided fertilizer production and reduced eutrophication potential. For example, nutrient removal efficiencies of 70–90% for nitrogen and phosphorus translate into eutrophication reductions of up to 60–90% compared to conventional activated sludge systems [80]. A major methodological challenge in LCA is the integration of wastewater treatment credits. Depending on the allocation method—system expansion, mass allocation, or economic allocation—the environmental performance of a given bioproduct can vary substantially. Recent assessments indicate that including wastewater treatment credits can reduce the GWP of microalgal biodiesel by 20–40%, improving the overall environmental performance of the system. However, these benefits remain highly sensitive to downstream processing efficiency [183,184,185].

#### 4.1.3. Comparative Methodological Challenges: Wastewater vs. Synthetic Media

Comparative analyses highlight that wastewater-derived biomass introduces methodological complexities not present in synthetic cultivation systems. Variability in nutrient loads, presence of heavy metals or organic contaminants, and seasonal fluctuations all influence both TEA and LCA outcomes [13,186]. For example, two studies evaluating carotenoid extraction from wastewater-grown microalgae reported purification-related energy demands differing by more than 50%, attributed to differences in contaminant profiles [187,188]. Such discrepancies underscore the need for standardized methodological frameworks that explicitly account for wastewater characteristics, contaminant removal requirements, and realistic scale-up assumptions [189]. Overall, TEA and LCA converge on a central conclusion: the feasibility of wastewater-derived microalgal bioproducts is governed by a small number of shared bottlenecks—harvesting efficiency, drying energy demand, biomass composition variability, and purification requirements [173]. While wastewater integration offers clear economic and environmental advantages through nutrient substitution and treatment credits, these benefits can only be realized if downstream processes are optimized and aligned with the intended bioproduct pathway [190]. Consequently, TEA and LCA must be applied jointly to capture the full spectrum of trade-offs and to guide the development of integrated microalgal biorefineries capable of producing economically viable and environmentally sustainable products [191].

### 4.2. Techno-Economic Performance and Value Chain Analysis of Wastewater Derived Microalgal Bioproducts

The techno-economic performance of microalgae-based wastewater systems is fundamentally determined by the balance between upstream cost reductions achieved through nutrient substitution and the downstream processing intensity required to convert biomass into valuable products. While wastewater provides a low-cost nutrient source and can reduce cultivation expenses by 30–70% compared to synthetic media, the economic feasibility of the entire system ultimately depends on the efficiency of harvesting, dewatering, and product extraction [173,192]. Recent studies emphasize that the economic viability of wastewater-derived microalgal bioproducts varies substantially across product categories, with biofuels generally performing poorly, biofertilizers and biogas showing moderate feasibility, and high-value compounds demonstrating the strongest economic potential [170,193].

Cultivation remains one of the most cost-sensitive stages, as reactor configuration strongly influences both capital requirements and achievable biomass productivity. Raceway ponds are widely regarded as the most cost-effective option due to their low construction costs and operational simplicity, whereas photobioreactors (PBRs) require substantially higher investment but can deliver two- to five-fold higher productivities under controlled conditions [194,195,196]. However, in wastewater-based systems, biomass productivity is typically lower and more variable than in synthetic media because of fluctuating nutrient loads, inhibitory compounds, and contamination risks [197]. Municipal wastewaters often yield 10–20 g m^−2^ day^−1^, while optimized synthetic media can reach 20–40 g m^−2^ day^−1^ [2]. Industrial wastewater may support higher growth due to elevated nutrient concentrations, but they also introduce contamination risks and may require pretreatment. These variations propagate downstream, influencing processing costs and final product yields [60,198]. Across TEA studies, harvesting and dewatering consistently emerge as the dominant operational and energetic bottlenecks, accounting for 20–60% of total OPEX [199,200]. Centrifugation provides high-purity biomass but is highly energy-intensive, often consuming 0.8–3.0 kWh kg^−1^ dry biomass, while flocculation–sedimentation reduces energy demand but may introduce chemical residues that complicate downstream valorization, especially for high-value products [2]. LCA studies confirm that inefficient harvesting can offset the environmental benefits of wastewater cultivation, as drying and centrifugation contribute 25–45% of total GWP and 40–80% of cumulative energy demand (CED). Consequently, harvesting is widely recognized as a shared TEA–LCA bottleneck and a primary determinant of system-level feasibility [201,202,203,204].

Downstream valorization pathways exhibit markedly different techno-economic and environmental profiles. Biodiesel remains economically uncompetitive due to low lipid content in wastewater-grown biomass and the high energy demand of extraction and conversion, with production costs typically 5–12 USD kg^−1^ and NER < 1 [205,206]. Biogas production offers a more favorable energy balance, with methane yields of 200–350 mL CH_4_ g^−1^ VS, and can offset internal energy demands, though revenue potential remains modest [179,207]. Biofertilizers combine strong environmental performance with favorable economics because nutrient-rich biomass requires minimal processing and can displace synthetic fertilizers, often reducing production costs by 20–50% relative to fuel-oriented pathways. High-value compounds provide the greatest revenue potential, with market prices ranging from 200 to >2000 USD kg^−1^, but require extensive purification when derived from wastewater biomass, increasing energy demand by 20–60% unless hybrid cultivation or advanced purification technologies are used [55,208,209,210].

## 5. Conclusions

Microalgae-based phycoremediation for wastewater treatment offers a highly sustainable, low-carbon alternative to conventional methods, effectively extracting nitrogen, phosphorus, and heavy metals while avoiding secondary chemical pollution. By shifting the operational paradigm from simple pollutant removal to resource recovery, wastewater streams serve as a cost-effective nutrient source for generating valuable biomass, which can be transformed into a diverse portfolio of circular bioproducts, including biofuels, biofertilizers, biostimulants, and high-value biopolymers. Fluctuating inputs cause biomass composition variability that impacts downstream processing yields. Furthermore, downstream harvesting, dewatering, and drying represent process hotspots, consuming 20–60% of operational costs and up to 80% of cumulative energy demand. Joint techno-economic analyses (TEA) and life cycle assessments (LCA) reveal that major socio-technical bottlenecks must be addressed before widespread commercial scaling is viable. Ultimately, single-product pathways remain economically uncompetitive, most notably for biofuel production; therefore, the future of the industry relies on integrated, multi-product biorefineries that simultaneously target high-value compound extraction alongside residual energy recovery to achieve a zero-waste process flow.

## Data Availability

No new data were created or analyzed in this study. Data sharing is not applicable to this article.

## References

[1] Cairone S., Hasan S.W., Choo K.-H., Lekkas D.F., Fortunato L., Zorpas A.A., Korshin G., Zarra T., Belgiorno V., Naddeo V. (2024). Revolutionizing wastewater treatment toward circular economy and carbon neutrality goals: Pioneering sustainable and efficient solutions for automation and advanced process control with smart and cutting-edge technologies. J. Water Process Eng..

[2] Calijuri M.L., Do Couto E.d.A., Assemany P.P., Ribeiro V.J., Lorentz J.F., Castro J.d.S., Assis L.R.d., Oliveira A.P.d.S., Pereira A.S.A.d.P., Marangon B.B. (2025). Microalgae-Based Wastewater Treatment and Biomass Valorization: Insights, Challenges, and Opportunities from 15 Years of Research. ACS Omega.

[3] Faragò M., Damgaard A., Madsen J.A., Andersen J.K., Thornberg D., Andersen M.H., Rygaard M. (2021). From wastewater treatment to water resource recovery: Environmental and economic impacts of full-scale implementation. Water Res..

[4] Ghimire U., Sarpong G., Gude V.G. (2021). Transitioning Wastewater Treatment Plants toward Circular Economy and Energy Sustainability. ACS Omega.

[5] Liu X.-Y., Hong Y. (2021). Microalgae-Based Wastewater Treatment and Recovery with Biomass and Value-Added Products: A Brief Review. Curr. Pollut. Rep..

[6] Zhang Y., Wang J.-H., Zhang J.-T., Chi Z.-Y., Kong F.-T., Zhang Q. (2023). The long overlooked microalgal nitrous oxide emission: Characteristics, mechanisms, and influencing factors in microalgae-based wastewater treatment scenarios. Sci. Total Environ..

[7] Teuma L., Sanz-Luque E., Guieysse B., Plouviez M. (2023). Are Microalgae New Players in Nitrous Oxide Emissions from Eutrophic Aquatic Environments?. Phycology.

[8] Mezzari M.P., da Silva M.L.B., Nicoloso R.S., Ibelli A.M.G., Bortoli M., Viancelli A., Soares H.M. (2013). Assessment of N_2_O emission from a photobioreactor treating ammonia-rich swine wastewater digestate. Bioresour. Technol..

[9] Matesun J., Petrik L., Musvoto E., Ayinde W., Ikumi D. (2024). Limitations of wastewater treatment plants in removing trace anthropogenic biomarkers and future directions: A review. Ecotoxicol. Environ. Saf..

[10] Moon S.R., Rahman M.M., Rahman A., Khan A.A., Nazir M.A., Islam M.A., Abdulla-Al-Mamun M. (2026). Water Resources and Environmental Sustainability: Current Challenges and Future Perspectives. Resources.

[11] Omokaro G.O., Nafula Z.S., Iloabuchi N.E., Chikukula A.A., Osayogie O.G., Nnoli E.C. (2025). Microalgae as biofactories for sustainable applications: Advancing carbon sequestration, bioenergy, and environmental remediation. Sustain. Chem. Clim. Action.

[12] Fal S., Smouni A., Arroussi H.E. (2023). Integrated microalgae-based biorefinery for wastewater treatment, industrial CO_2_ sequestration and microalgal biomass valorization: A circular bioeconomy approach. Environ. Adv..

[13] Abdelfattah A., Ali S.S., Ramadan H., El-Aswar E.I., Eltawab R., Ho S.-H., Elsamahy T., Li S., El-Sheekh M.M., Schagerl M. (2023). Microalgae-based wastewater treatment: Mechanisms, challenges, recent advances, and future prospects. Environ. Sci. Ecotechnology.

[14] Amaro H.M., Salgado E.M., Nunes O.C., Pires J.C.M., Esteves A.F. (2023). Microalgae systems—Environmental agents for wastewater treatment and further potential biomass valorisation. J. Environ. Manag..

[15] Sati S., Sharma P.K., Naithani P., Jha P.K., Karmakar R., Behera N.R. (2026). Nature-based wastewater solutions: A comprehensive review of algae–wetland integration for nutrient and microplastics removal. Front. Water.

[16] Bulynina S.S., Ziganshina E.E., Terentev A.D., Ziganshin A.M. (2026). Treatment of Wastewater from the Fish Processing Industry and Production of Valuable Algal Biomass with a Biostimulating Effect. Phycology.

[17] Plöhn M., Spain O., Sirin S., Silva M., Escudero-Oñate C., Ferrando-Climent L., Allahverdiyeva Y., Funk C. (2021). Wastewater treatment by microalgae. Physiol. Plant..

[18] Fernandes J., Ramísio P.J., Puga H. (2024). A Comprehensive Review on Various Phases of Wastewater Technologies: Trends and Future Perspectives. Eng.

[19] Agarwal S., Darbar S., Saha S., Srivastav A.L., Madhav S., Bhardwaj A.K., Valsami-Jones E. (2022). Chapter 25—Challenges in management of domestic wastewater for sustainable development. Current Directions in Water Scarcity Research.

[20] Purba L.D.A., Effendi D.B., Kurniawan K.I.A., Setiawan A.A.R., Kamyab H., Yuzir A., Susanti H. (2026). Sustainable production of aviation biofuel using wastewater-grown microalgae: Process optimization and life cycle assessment of scenario-based strategies. Fuel.

[21] Van D.T.C., Dung N.T.P., Uyen B.T.T., Thuan T.D., Giang L.T. (2026). Integrated valorization of domestic wastewater for phycoremediation and polyhydroxybutyrate (PHB) production by Synechocystis salina M8. Bioresour. Technol. Rep..

[22] Xu K., Yu H., Zhu Z., Shen Z., Zou X. (2025). Sustainable nutrient removal and biomass production in a self-permeation microalgae biofilm system with mechanistic and life cycle perspectives. J. Environ. Chem. Eng..

[23] Okut N., Hamzat A.K., Rajakaruna R.A.D.N.V., Asmatulu E. (2025). Agricultural wastewater treatment and reuse technologies: A comprehensive review. J. Water Process Eng..

[24] Kong T., Li A., Zhang L. (2025). Microalgae-based wastewater treatment: Mechanisms, strategies, and the role of biochemical composition. J. Environ. Chem. Eng..

[25] Ali S.S., Fodah A.E.M., Jiao H., Ahmad I., Liu J. (2026). Microalgae-based wastewater treatment and carbon dioxide capture: From mechanisms to scalable circular bioeconomy systems. Process Saf. Environ. Prot..

[26] Ramsuroop J., Gutu L., Ayinde W.B., Basitere M., Manono M.S. (2024). A Review of Biological Processes for Dairy Wastewater Treatment and the Effect of Physical Parameters Which Affect Their Efficiency. Water.

[27] Krishnamoorthy S., Rajamanickam R., Mahmoud A.E.D., Saleh O., Fatriasari W., Selvasembian R. (2026). Dairy wastewater treatment technologies and their valorization via stress-induced microalgae cultivation for lipid accumulation. Bioresour. Technol. Rep..

[28] Paulenco A., Vintila A.C.N., Vlaicu A., Ciltea-Udrescu M., Galan A.-M. (2023). *Nannochloris* sp. Microalgae Strain for Treatment of Dairy Wastewaters. Microorganisms.

[29] Gălan A.-M., Vlaicu A., Vintilă A.C.N., Cîlţea-Udrescu M., Cerchezan G., Frone A.N., Vasilievici G., Paulenco A. (2022). Microalgae Strain Porphyridium purpureum for Nutrient Reduction in Dairy Wastewaters. Sustainability.

[30] Phyu K.K., Zhi S., Liang J., Yang Z., Zhao R., Liu J., Cao Y., Wang H., Zhang K. (2026). Biomass growth, nutrient removal, and microbial community dynamics in mono-, Co-, and sequential culture of screened cyanobacteria with microalgae for dairy wastewater treatment. Bioresour. Technol..

[31] Xiao R., Carter E., Allen A., Tan P.-L., Zheng Y.-H., Chen Q., Zhu S.-N., Popat S.C., Knopf A., Williams C.F. (2025). Nutrient recovery from high-salinity dairy wastewater through the cultivation of acclimatized microalgae. J. Water Process Eng..

[32] Zhang M., Xin R., Wang Y., Zhang K. (2025). Screening and characterization of marine cyanobacteria for dairy farm wastewater treatment: *Nodosilinea* sp. E11 as a promising candidate for phycoremediation. J. Clean. Prod..

[33] Mahdavi S., Tavakoli O. (2025). Utilizing co-cultivation of Tetraselmis chuii and *Chlorella vulgaris* to improve high-value biomass yield in dairy wastewater treatment. J. Water Process Eng..

[34] Gonzales Cruz C., Centeno da Rosa A.P., Strentzle B.R., Vieira Costa J.A. (2023). Microalgae-based dairy effluent treatment coupled with the production of agricultural biostimulant. J. Appl. Phycol..

[35] Virpiranta H., Okyere Abayie S., Mäkikangas J., Puirava M., Koivula K., Leiviskä T. (2023). Treatment of fish processing plant wastewater using dissolved air flotation and pilot-scale biochar column filtration. J. Environ. Chem. Eng..

[36] Al-Dawery S.K., AL-Yaqoubi G.E., Al-Musharrafi A.A., Harharah H.N., Amari A., Harharah R.H. (2023). Treatment of Fish-Processing Wastewater Using Polyelectrolyte and Palm Anguish. Processes.

[37] Khalatbari S., Sotaniemi V.-H., Suokas M., Taipale S., Leiviskä T. (2024). Microalgae technology for polishing chemically-treated fish processing wastewater. Groundw. Sustain. Dev..

[38] Christodoulou X., M’Ahmed C., Zili F., Bessadok B., Sadok S., Monney I., Rothlisberger R., Bagnoud M. (2025). Design and development of pilot photobioreactor for simultaneous microalgae cultivation and aquaculture wastewater treatment. Process Biochem..

[39] Zhao C., Lu B., Wang Z., Wei J., Zhao Y., Wang S. (2025). Nano zero-valent iron mediated optimization of microalgae-based systems for efficient nutrient and antibiotics removal from aquaculture wastewater. Int. Biodeterior. Biodegrad..

[40] Nagarajan D., Kusmayadi A., Yen H.-W., Dong C.-D., Lee D.-J., Chang J.-S. (2019). Current advances in biological swine wastewater treatment using microalgae-based processes. Bioresour. Technol..

[41] Wang C., Wang H., Zheng R., Liu D., Zhang Q., Lu H., Wang X., Liu D. (2025). Piggery wastewater treatment by microalgae: Mechanisms and technologies. Algal Res..

[42] He J., Zhang J., Ren H., Zhang Y., Li H., Liu H. (2025). Carbon dioxide-assisted enhancement of microalgae growth and pollutant removal in piggery wastewater by newly-isolated ammonia-tolerant microalgae *Chlorella sorokinfana*. Algal Res..

[43] Flores-Zambrano K., Tapia W., Castillejo P. (2025). Microalgae strains isolated from piggery wastewater in Ecuador: Effective nitrogen compound removal and growth potential in extremophile conditions. Biotechnol. Rep..

[44] Thi Cam Van D., Thi Mai D., Thi Thu Uyen B., Thi Phuong Dung N., Thi Thu Ha L., Thi Lieu N., Minh D.N., Thuan T.D., Giang L.T. (2025). Sustainable remediation of piggery wastewater using a novel mixotrophic *Chlorella sorokiniana* Cbeo for high value biomass production. Biochem. Eng. J..

[45] Rossi S., Pizzera A., Bellucci M., Marazzi F., Mezzanotte V., Parati K., Ficara E. (2022). Piggery wastewater treatment with algae-bacteria consortia: Pilot-scale validation and techno-economic evaluation at farm level. Bioresour. Technol..

[46] Wang B., Zhang L., Lian L., Zhang X., Qi Y. (2025). Treatment of compound pollution in simulated livestock and poultry wastewater by algae-bacteria symbiosis system. Chemosphere.

[47] Ummalyma S.B., Chiang A., Herojit N., Arumugam M. (2023). Sustainable microalgal cultivation in poultry slaughterhouse wastewater for biorefinery products and pollutant removal. Bioresour. Technol..

[48] Khiewwijit R., Chainetr S., Thiangchanta S., Ngoenkhumkhong K. (2024). Development of sustainable poultry waste management using integrated microalgae cultivation: Towards performance, resource recovery and environmental impact. Heliyon.

[49] Devrajani S.K. (2025). A sustainable microalgal cultivation approach for the treatment of poultry abattoir wastewater and biofuel production. Environ. Monit. Assess..

[50] Kato S., Kansha Y. (2024). Comprehensive review of industrial wastewater treatment techniques. Environ. Sci. Pollut. Res..

[51] Mohd Udaiyappan A.F., Abu Hasan H., Takriff M.S., Sheikh Abdullah S.R. (2017). A review of the potentials, challenges and current status of microalgae biomass applications in industrial wastewater treatment. J. Water Process Eng..

[52] Javed F., Rehman F., Khan A.U., Fazal T., Hafeez A., Rashid N. (2022). Real textile industrial wastewater treatment and biodiesel production using microalgae. Biomass Bioenergy.

[53] Younas M., Rehman F., Zuhair S.A., Ahmed F., Muzafar M., Awad A., Asif M., Javed F. (2025). Synergistic approach to industrial wastewater treatment: Combining plasmolysis and microalgae cultivation. Chem. Eng. Process.-Process Intensif..

[54] Abate R., Oon Y.-S., Oon Y.-L., Bi Y. (2024). Microalgae-bacteria nexus for environmental remediation and renewable energy resources: Advances, mechanisms and biotechnological applications. Heliyon.

[55] Alavianghavanini A., Shayesteh H., Bahri P.A., Vadiveloo A., Moheimani N.R. (2024). Microalgae cultivation for treating agricultural effluent and producing value-added products. Sci. Total Environ..

[56] Zhang H., Wu Y., Liu D., Feng S., Xuan X., Dong G., Cheng J., Qin Y., Ngo H.H. (2025). Insights into microalgal biotechnology: Current applications, key challenges, and future prospects. J. Environ. Manag..

[57] Ma X., Jian W. (2023). Growth Conditions and Growth Kinetics of *Chlorella vulgaris* Cultured in Domestic Sewage. Sustainability.

[58] Sousa S.A., Machado C.A., Esteves A.F., Salgado E.M., Dias J.M., Vilaça J.S., Pires J.C.M. (2025). Microalgae-based wastewater remediation: Linking N:P ratio and nitrogen sources to treatment performance by *Chlorella vulgaris* and biomass valorisation. Chem. Eng. J..

[59] Kong Q.-X., Li L., Martinez B., Chen P., Ruan R. (2010). Culture of Microalgae Chlamydomonas reinhardtii in Wastewater for Biomass Feedstock Production. Appl. Biochem. Biotechnol..

[60] Pant S., Goel M., Sahoo N.K., Yuan Q., Rout P.R. (2025). Microalgae-based treatment for wastewater management and valorization. Curr. Opin. Environ. Sci. Health.

[61] Zhou S., Li W., He S. (2023). Microalgal diversity enhances water purification efficiency in experimental microcosms. Front. Ecol. Evol..

[62] Shahid A., Rehman A.U., Usman M., Ashraf M.U.F., Javed M.R., Khan A.Z., Gill S.S., Mehmood M.A. (2020). Engineering the metabolic pathways of lipid biosynthesis to develop robust microalgal strains for biodiesel production. Biotechnol. Appl. Biochem..

[63] Inwongwan S., Sriwari S., Pumas C. (2025). Metabolomic Insights into the Adaptations and Biotechnological Potential of Euglena gracilis Under Different Trophic Conditions. Plants.

[64] Praveen K., Abinandan S., Venkateswarlu K., Megharaj M. (2024). Harnessing Extremophilic Trait and Metabolic Flexibility of Microalgal Strains for the Treatment of Highly Alkaline Winery Wastewater. ACS ES T Eng..

[65] Zhou D., Zhang Q., Wang H., He X., Qv M., Zong Y., Zhu L. (2025). Spontaneous reshaping of microalgae carbon metabolism under organophosphate esters stress: Insights into adaptive strategies for wastewater treatment. Bioresour. Technol..

[66] Atatoprak-Gonçalves T., Esteves B., Cruz-Lopes L. (2026). Microalgae-Based Treatment of Cheese Whey Wastewater for Circular Bioeconomy Applications. Sustainability.

[67] Wollmann F., Dietze S., Ackermann J.U., Bley T., Walther T., Steingroewer J., Krujatz F. (2019). Microalgae wastewater treatment: Biological and technological approaches. Eng. Life Sci..

[68] Al-Jabri H., Das P., Khan S., Thaher M., AbdulQuadir M. (2021). Treatment of Wastewaters by Microalgae and the Potential Applications of the Produced Biomass—A Review. Water.

[69] Barone R., De Napoli L., Mayol L., Paolucci M., Volpe M.G., D’Elia L., Pollio A., Guida M., Gambino E., Carraturo F. (2020). Autotrophic and Heterotrophic Growth Conditions Modify Biomolecole Production in the Microalga Galdieria sulphuraria (Cyanidiophyceae, Rhodophyta). Mar. Drugs.

[70] Su Y. (2021). Revisiting carbon, nitrogen, and phosphorus metabolisms in microalgae for wastewater treatment. Sci. Total Environ..

[71] Kupriyanova E.V., Pronina N.A., Los D.A. (2023). Adapting from Low to High: An Update to CO_2_-Concentrating Mechanisms of Cyanobacteria and Microalgae. Plants.

[72] Hariz H.B., Takriff M.S., Mohd Yasin N.H., Ba-Abbad M.M., Mohd Hakimi N.I.N. (2019). Potential of the microalgae-based integrated wastewater treatment and CO_2_ fixation system to treat Palm Oil Mill Effluent (POME) by indigenous microalgae; *Scenedesmus* sp. and *Chlorella* sp.. J. Water Process Eng..

[73] Razzak S.A. (2025). Effect of temperature and CO_2_ concentration on biological nutrient removal from tertiary municipal wastewater using microalgae *Chlorella prototheocoides*. Biotechnol. Notes.

[74] Li X., Chen J., Liu X., Wang F. (2026). Photocatalysis-microalgae coupled system for ciprofloxacin-containing agricultural wastewater treatment: Pollutant removal, carbon fixation and synergistic relationship. J. Hazard. Mater..

[75] Moghazy R.M., Abdalla S.B. (2024). Boosting wastewater treatment and CO_2_ bioremediation with Nile River microalgae: Resilience to simulated smoke and enhanced biomass production. Bioresour. Technol. Rep..

[76] Gill J.M., Hussain S.M., Ali S., Zahoor A.F., Alasmari A., Munir M., Naeem E., Amjad M., Yousaf Z., Faisal M. (2025). Optimizing aquaculture sustainability: Microalgae-based co-culture systems for aquaculture wastewater treatment and pollution reduction. Algal Res..

[77] Mohsenpour S.F., Hennige S., Willoughby N., Adeloye A., Gutierrez T. (2021). Integrating micro-algae into wastewater treatment: A review. Sci. Total Environ..

[78] Sun Q., Zhang X., Zhang X. (2023). Impact of Natural Microorganisms on the Removal of COD and the Cells Activity of the *Chlorella* sp. in Wastewater. Water.

[79] Cai T., Park S.Y., Li Y. (2013). Nutrient recovery from wastewater streams by microalgae: Status and prospects. Renew. Sustain. Energy Rev..

[80] Popa M.D., Simionov I.-A., Petrea S.M., Georgescu P.-L., Ifrim G.A., Iticescu C. (2025). Efficiency of Microalgae Employment in Nutrient Removal (Nitrogen and Phosphorous) from Municipal Wastewater. Water.

[81] Najar-Almanzor C.E., Velasco-Iglesias K.D., Solis-Bañuelos M., González-Díaz R.L., Guerrero-Higareda S., Fuentes-Carrasco O.J., García-Cayuela T., Carrillo-Nieves D. (2024). *Chlorella vulgaris*-mediated bioremediation of food and beverage wastewater from industries in Mexico: Results and perspectives towards sustainability and circular economy. Sci. Total Environ..

[82] Alazaiza M.Y.D., He S., Su D., Abu Amr S.S., Toh P.Y., Bashir M.J.K. (2023). Sewage Water Treatment Using *Chlorella vulgaris* Microalgae for Simultaneous Nutrient Separation and Biomass Production. Separations.

[83] Zieliński M., Kisielewska M., Talpalaru A., Rusanowska P., Kazimierowicz J., Dębowski M. (2025). Integration of Aquaculture Wastewater Treatment and *Chlorella vulgaris* Cultivation as a Sustainable Method for Biofuel Production. Energies.

[84] Rani S., Ojha C.S.P. (2021). *Chlorella sorokiniana* for integrated wastewater treatment, biomass accumulation and value-added product estimation under varying photoperiod regimes: A comparative study. J. Water Process Eng..

[85] Lugo L.A., Thorarinsdottir R.I., Bjornsson S., Palsson O.P., Skulason H., Johannsson S., Brynjolfsson S. (2020). Remediation of Aquaculture Wastewater Using the Microalga *Chlorella sorokiniana*. Water.

[86] Ruan L., Cheng M., Xu D., Wu L., Liang Y., Zhang X., Zhang T., Huang Y., Guo C., Shang C. (2024). Nutrient Removal and Lipid Production Using *Chlorella pyrenoidosa* in Unsterilized Domestic Wastewater. Waste Biomass Valorization.

[87] Calatrava V., Hom E.F.Y., Llamas Á., Fernández E., Galván A. (2019). Nitrogen scavenging from amino acids and peptides in the model alga Chlamydomonas reinhardtii. The role of extracellular l-amino oxidase. Algal Res..

[88] Liu J., Wu Y., Wu C., Muylaert K., Vyverman W., Yu H.-Q., Muñoz R., Rittmann B. (2017). Advanced nutrient removal from surface water by a consortium of attached microalgae and bacteria: A review. Bioresour. Technol..

[89] Göncü S., Şimşek Uygun B., Atakan S. (2025). Nitrogen and phosphorus removal from wastewater using *Chlorella vulgaris* and *Scenedesmus quadricauda* microalgae with a batch bioreactor. Int. J. Environ. Sci. Technol..

[90] Laabassi A., Fercha A., Bellucci S., Postiglione A., Maresca V., Dentato M., Boudehane A., Amira L., Saada F.Z., Boukehil R. (2026). Phosphorus Loading Drives Microalgal Community Changes and Enhances Nutrient Removal in Photobioreactors Treating Synthetic Wastewater. Plants.

[91] Salsali H., McBean E. (2014). Nutrient removal (nitrogen and phosphorous) in secondary effluent from a wastewater treatment plant by microalgae. Can. J. Civ. Eng..

[92] Machado A.A., Valiaparampil J.G., M L. (2024). Unlocking the Potential of Algae for Heavy Metal Remediation. Water Air Soil Pollut..

[93] Leon-Miranda E., Tejada-Jimenez M., Llamas A. (2025). Insertional Mutagenesis as a Strategy to Open New Paths in Microalgal Molybdenum and Nitrate Homeostasis. Curr. Issues Mol. Biol..

[94] Tejada-Jimenez M., Leon-Miranda E., Llamas A. (2023). Chlamydomonas reinhardtii—A Reference Microorganism for Eukaryotic Molybdenum Metabolism. Microorganisms.

[95] Mungunkhuyag K., Steingroewer J., Walther T., Krujatz F. (2026). Isolation and characterization of heavy metal tolerant microalgae from old mining areas of Saxony. Sci. Rep..

[96] Huang Y., Yu J., Huang X., Sun J., Tang W., Wei M., Yang G., Fu Y. (2025). Algal-bacterial symbiotic system for treatment of heavy metal containing wastewater: Performance, mechanisms and applications. npj Clean Water.

[97] Liu S., Kong Z., Guo H., Zhang Y., Han X., Gao Y., Daigger G.T., Zhang G., Li R., Liu Y. (2024). Performance, mechanism regulation and resource recycling of bacteria-algae symbiosis system for wastewater treatment: A review. Environ. Pollut..

[98] Nguyen T.-M.-P., Nguyet P.N., Long N.H., Nguyen P.-T., Nguyen H.-V., Tran C.-S., Du M.-L., Hatamoto M., Watari T., Yamaguchi T. (2026). Enhanced nutrient removal and microbial stability in a hybrid membrane bioreactor driven by *Chlorella sorokiniana*-Bacteria synergism. J. Water Process Eng..

[99] Sátiro J., Cunha A., Gomes A.P., Simões R., Albuquerque A. (2022). Optimization of Microalgae–Bacteria Consortium in the Treatment of Paper Pulp Wastewater. Appl. Sci..

[100] Llamas A., Leon-Miranda E., Tejada-Jimenez M. (2023). Microalgal and Nitrogen-Fixing Bacterial Consortia: From Interaction to Biotechnological Potential. Plants.

[101] Palikrousis T.L., Manolis C., Kalamaras S.D., Samaras P. (2024). Effect of Light Intensity on the Growth and Nutrient Uptake of the Microalga *Chlorella sorokiniana* Cultivated in Biogas Plant Digestate. Water.

[102] Tian X., Lin X., Xie Q., Liu J., Luo L. (2024). Effects of Temperature and Light on Microalgal Growth and Nutrient Removal in Turtle Aquaculture Wastewater. Biology.

[103] Venkata Subhash G., Rohit M.V., Devi M.P., Swamy Y.V., Venkata Mohan S. (2014). Temperature induced stress influence on biodiesel productivity during mixotrophic microalgae cultivation with wastewater. Bioresour. Technol..

[104] Xu K., Zou X., Wen H., Xue Y., Qu Y., Li Y. (2019). Effects of multi-temperature regimes on cultivation of microalgae in municipal wastewater to simultaneously remove nutrients and produce biomass. Appl. Microbiol. Biotechnol..

[105] Yu H., Kim J., Rhee C., Shin J., Shin S.G., Lee C. (2022). Effects of Different pH Control Strategies on Microalgae Cultivation and Nutrient Removal from Anaerobic Digestion Effluent. Microorganisms.

[106] Beltrán-Rocha J.C., Guajardo-Barbosa C., Barceló-Quintal I.D., Reyna-Martínez G., Fariz-Salinas E., Ramírez-Castillo A., Rodríguez-Fuentes H., López-Chuken U.J. (2024). Effect of natural increase of pH and microalgae cyclical re-cultivation on biomass production and polishing of municipal secondary effluent. Desalin. Water Treat..

[107] Kalwani M., Vítová M. (2026). Artificial Intelligence–driven microalgal biorefineries: Advancing wastewater phycoremediation toward sustainable biofuel and high-value product generation. Algal Res..

[108] Bora A., Thondi Rajan A.S., Ponnuchamy K., Muthusamy G., Alagarsamy A. (2024). Microalgae to bioenergy production: Recent advances, influencing parameters, utilization of wastewater—A critical review. Sci. Total Environ..

[109] de Lima Barizão A.C., de Oliveira Gomes L.E., Brandão L.L., Sampaio I.C.F., de Moura I.V.L., Gonçalves R.F., de Oliveira J.P., Cassini S.T. (2023). Microalgae as tertiary wastewater treatment: Energy production, carbon neutrality, and high-value products. Algal Res..

[110] Zhang C., Chen X., Han M., Li X., Chang H., Ren N., Ho S.H. (2023). Revealing the role of microalgae-bacteria niche for boosting wastewater treatment and energy reclamation in response to temperature. Environ. Sci. Ecotechnology.

[111] de Andrade F.P., de Farias Silva C.E., Medeiros J.A., Vieira R.C., de Sá Filho M.L.F., Santos G.K.S. (2022). Consortium between microalgae and other microbiological groups: A promising approach to emphasise the sustainability of open cultivation systems for wastewater treatment. J. Water Process Eng..

[112] Zhang H., Xu B., Zhao C., Liu J., Zhao Y., Sun S., Wei J. (2022). Simultaneous biogas upgrading and biogas slurry treatment by different microalgae-based technologies under various strigolactone analog (GR24) concentrations. Bioresour. Technol..

[113] Padri M., Boontian N., Teaumroong N., Piromyou P., Piasai C. (2022). Application of Aspergillus niger F5 as an alternative technique to harvest microalgae and as a phosphorous removal treatment for cassava biogas effluent wastewater. J. Water Process Eng..

[114] Marangon B.B., Castro J.S., Ballotin F.C., Silva L.S., Assemany P., do Couto E.A., Silva T.A., Jesus Junior M.M., Ribeiro V.J., Ribeiro Junior J.I. (2026). One-step Hydrothermal Liquefaction and Catalytic Upgrading of Wastewater-Grown Microalgae for Potential Sustainable Aviation Fuel Precursors. ACS Omega.

[115] Kumar A., Watkins J.D., Cronin D., Schmidt A.J., Santosa D.M., Yang Z., Heyne J., Valdez P.J. (2025). Hydrothermal liquefaction of wastewater-grown algae to produce synthetic aviation fuel: A combined experimental study and techno-economic assessment. Energy Convers. Manag. X.

[116] Rojas M., Manrique R., Hornung U., Funke A., Mullen C.A., Chejne F., Maya J.C. (2025). Advances and challenges on hydrothermal processes for biomass conversion: Feedstock flexibility, products, and modeling approaches. Biomass Bioenergy.

[117] Rivas-Navia D., CheikhWafa J., Zurita A., Torrens E., Bengoa C. (2025). Design of a Ɛ-caprolactam-based ionic liquids for the extraction of lipids from starved Parachlorella kessleri microalgae for sustainable aviation fuels (SAF). Algal Res..

[118] Singh P., Mohanty K. (2026). Bio-oil as a Promising Product from Co-liquefaction of Dairy Wastewater Grown Microalgae with Dairy Sludge: Study on Synergistic Effect and Sustainable Energy Generation. Renew. Energy.

[119] Hassan S.H., Attia N.K., El Diwani G.I., Amin S.K., Abdo S.M., Ashour F.H., Abadir E.F. (2026). Parametric study and fuel quality assessment of biofuel from hydrothermal liquefaction of microalgae grown in municipal wastewater. Sci. Rep..

[120] Silva T.A., Magno de Jesus Junior M., Magalhães I.B., Ananias M.S., Aona de Paula Pereira A.S., de Ávila Rodrigues F., Delgado dos Reis A.J., Calijuri M.L. (2025). Bio-oil from hydrothermal liquefaction of microalgae cultivated in wastewater: An economic and life cycle approach. J. Clean. Prod..

[121] Oliveira A.P.S., Assemany P.P., Jackeline de Siqueira C., de Jesus Junior M.M., Rodrigues F.A., de Oliveira L.F.C., Campos M.T.C., Oliveira Carneiro A.C., Calijuri M.L. (2025). Hydrothermal Carbonization of Microalgae Biomass from Wastewater Treatment: Effects of Acid Pretreatment. ACS Omega.

[122] Al Shehhi A., Souissi Y., Nair A.S., Usmani Z., Sharma M., Sivakumar N. (2025). Microbial lipid-based biodiesel production using wastewater: Opportunities and challenges. Bioresour. Bioprocess..

[123] Sharma A.K., Jaryal S., Sharma S., Dhyani A., Tewari B.S., Mahato N. (2025). Biofuels from Microalgae: A Review on Microalgae Cultivation, Biodiesel Production Techniques and Storage Stability. Processes.

[124] Hoang A.T., Sirohi R., Pandey A., Nižetić S., Lam S.S., Chen W.-H., Luque R., Thomas S., Arıcı M., Pham V.V. (2022). Biofuel production from microalgae: Challenges and chances. Phytochem. Rev..

[125] Jivani F., Patwardhan S., Shinde A., Nayak M., Guldhe A. (2026). Process-intensified in-situ transesterification of wastewater-grown Marvania coccoides biomass using immobilized lipase for biodiesel production. Chem. Eng. Process.-Process Intensif..

[126] Leong W.H., Lim J.W., Rawindran H., Liew C.S., Lam M.K., Ho Y.C., Khoo K.S., Kusakabe K., Abdelghani H.T.M., Ho C.D. (2023). Energy balance and life cycle assessments in producing microalgae biodiesel via a continuous microalgal-bacterial photobioreactor loaded with wastewater. Chemosphere.

[127] de Mendonca H.V., Otenio M.H., Marchao L., Lomeu A., de Souza D.S., Reis A. (2022). Biofuel recovery from microalgae biomass grown in dairy wastewater treated with activated sludge: The next step in sustainable production. Sci. Total Environ..

[128] Kumar V., Gururani P., Parveen A., Verma M., Kim H., Vlaskin M., Grigorenko A.V., Rindin K.G. (2022). Dairy Industry wastewater and stormwater energy valorization: Effect of wastewater nutrients on microalgae-yeast biomass. Biomass Convers. Biorefinery.

[129] Huang H., Zhong S., Wen S., Luo C., Long T. (2022). Improving the efficiency of wastewater treatment and microalgae production for biofuels. Resour. Conserv. Recycl..

[130] Ziganshina E.E., Bulynina S.S., Yureva K.A., Ziganshin A.M. (2022). Growth Parameters of Various Green Microalgae Species in Effluent from Biogas Reactors: The Importance of Effluent Concentration. Plants.

[131] Li G., Hu R., Wang N., Yang T., Xu F., Li J., Wu J., Huang Z., Pan M., Lyu T. (2022). Cultivation of microalgae in adjusted wastewater to enhance biofuel production and reduce environmental impact: Pyrolysis performances and life cycle assessment. J. Clean. Prod..

[132] Santurbano V., Marangon B., Castro J., Calijuri M.L., Leme M., Assemany P. (2024). Enhancing environmental performance in biogas production from wastewater-grown microalgae: A life cycle assessment perspective. J. Environ. Manag..

[133] Barros R., Raposo S., Morais E.G., Rodrigues B., Afonso V., Gonçalves P., Marques J., Cerqueira P.R., Varela J., Teixeira M.R. (2022). Biogas Production from Microalgal Biomass Produced in the Tertiary Treatment of Urban Wastewater: Assessment of Seasonal Variations. Energies.

[134] Çınar A., Koca Akkaya E. (2026). Optimization of Ultrasound Pretreatment Applications in Biogas Production from Microalgae with the Taguchi Approach. BioEnergy Res..

[135] Javed M.A., Hassan A.A. (2026). Decoupling methanogenesis to boost hydrogen production via microalgae–wastewater co-digestion in a continuous stirred reactor. Fuel.

[136] Kurniawan K.I.A., Susanti H., Rani D.S., Harahap B.M., Firmansyah E.A., Ishizaki R., Demura M., Ahamed T., Noguchi R. (2024). Techno-economic analysis of biocrude, biogas, and fertilizer production from microalgae Coelastrella striolata cultivated in agroindustrial wastewater. J. Clean. Prod..

[137] Silva T.A., de Araujo M.N., de Rezende E.G.F., Saia F.T., Magalhães I.B., de Paula Pereira A.S.A., Ferreira J., Gregoracci G.B., Tallarico Adorno M.Â., Fuess L.T. (2025). Biohydrogen production from wastewater-grown microalgae-bacteria consortia: Optimizing inoculum selection for enhanced yield. Renew. Energy.

[138] Ruales E., Bellver M., Alvarez-Gonzalez A., Carvalho Fontes Sampaio I., Garfi M., Ferrer I. (2025). Carotenoids and biogas recovery from microalgae treating wastewater. Bioresour. Technol..

[139] Condor B.E., de Luna M.D.G., Lacson C.F.Z., Acebu P.I.G., Abarca R.R.M., Nagarajan D., Lee D.-J., Chang J.-S. (2024). Effects of carbon dioxide concentration and swine wastewater on the cultivation of *Chlorella vulgaris* FSP-E and bioethanol production from microalgae biomass. Appl. Energy.

[140] Ragul R., Ramesh P., Ganeshmani A., Shunmugam S., Manickavasagam M., Thajuddin N., Koventhan C., Muralitharan G., Lo A.-Y. (2026). Integrating Cyanobacterial Biomass, Biodiesel, and Bioethanol Production During Municipal Wastewater Treatment Towards a Cleaner and Greener Approach. Environments.

[141] Loni S., Shinde A., Kshirsagar R., Tanwar M., Guldhe A. (2026). Integration approach of microalgae cultivation with biomethanation derived wastewater as nutrient source for sustainable bioethanol production. Bioresour. Technol..

[142] Ngerem E.C., Sanusi I.A., Kana G.E.B., Olaniran A.O. (2025). Optimization of co-valorisation techniques for dairy and paper pulp wastewater in the cultivation of *Chlorococcum* sp. with a focus on mixture design, microwave-assisted pretreatment, and bioethanol production. Heliyon.

[143] Deshmukh M., Pathan A. (2026). An eco-friendly solution for bioethanol production from microalgae with optimized process parameters and emission characteristics. Renew. Sustain. Energy Rev..

[144] Iakovidou G., Itziou A., Tsiotsias A., Lakioti E., Samaras P., Tsanaktsidis C., Karayannis V. (2024). Application of Microalgae to Wastewater Bioremediation, with CO_2_ Biomitigation, Health Product and Biofuel Development, and Environmental Biomonitoring. Appl. Sci..

[145] Onay M. (2026). Biobutanol and carotenoid production from Hindakia tetrachotoma grown in microplastic-contaminated wastewater within a biorefinery concept. Alex. Eng. J..

[146] Pathania R., Saxena P., Sahoo S., Kundu A. (2026). Bioethanol and biobutanol production using algal biomass: Pathways and industrial applications. Front. Fuels.

[147] Brito-Lopez C., van der Wielen N., Barbosa M., Karlova R. (2025). Plant growth-promoting microbes and microalgae-based biostimulants: Sustainable strategy for agriculture and abiotic stress resilience. Philos. Trans. R. Soc. B Biol. Sci..

[148] Alvarez-Gonzalez A., Serrano L., Gorchs G., Uggetti E. (2025). Exploring the biostimulant potential of *Scenedesmus* sp. grown in wastewater: Impacts on plant growth and photosynthetic activity of lettuce. Chemosphere.

[149] Jesus M.M., Castro J.S., Marangon B.B., Magalhães I.B., Ribeiro V.J., Gama R.C.N., Rodrigues F.A., Calijuri M.L. (2025). From wastewater to fields: Exploring the scale-up and economic viability of biofertilizer production from microalgae cultivated in wastewater. J. Environ. Chem. Eng..

[150] Alvarez-Gonzalez A., Greque de Morais E., Planas-Carbonell A., Uggetti E. (2023). Enhancing sustainability through microalgae cultivation in urban wastewater for biostimulant production and nutrient recovery. Sci. Total Environ..

[151] Mesa A.P., Grattz P.A.C., Vargas J.J.V., Ríos L.A., Echeverri D.O., Parra A.M.M. (2025). Feasibility of nitrogen and phosphorus removal from treated wastewater using microalgae and potential microalgae use as biofertilizer. J. Water Process Eng..

[152] Akuku V., Satognon F. (2025). Sustainable phosphorus recovery from wastewater using microalgae: Economic, environmental, and agronomic implications for future phosphorus fertilizer solutions. Clean. Waste Syst..

[153] Castro I.M.P., Rosa A., Borges A., Cunha F., Passos F. (2024). The effects of microalgae use as a biofertilizer on soil and plant before and after its anaerobic (co-)digestion with food waste. Sci. Total Environ..

[154] Ruales E., Gómez-Serrano C., Morillas-España A., Garfí M., González-López C.V., Ferrer I. (2025). Microalgae-based biorefinery for biostimulant and biogas production: An integrated approach for resource recovery. Algal Res..

[155] Faliagka S., Kountrias G., Dimitriou E., Álvarez-Gil M., Blanco-Vieites M., Magrassi F., Notari M., Pechlivani E.M., Katsoulas N. (2024). Development of a Greenhouse Wastewater Stream Utilization System for On-Site Microalgae-Based Biostimulant Production. AgriEngineering.

[156] Assemany P., de Souza I.L., Muchico J.d.M.J., Scalco K.M.F.C., Balbino M.V.M., Santurbano V., Nascimento L.C.d.P., de Oliveira O.N., Siniscalchi L.A.B. (2025). Microalgae biotechnology applied to wastewater treatment and biopolymer production: A sustainable alternative for resource recovery. J. Water Process Eng..

[157] Xu P., Wang Y., Luo C., Xue A., Du H., Chen J. (2026). Advances in Synthetic Strategies for Microalgal Carotenoid Enhancement and Emerging Applications. Antioxidants.

[158] Liang M.H., Li C.P., Zhang W.P., Wang D.Z. (2026). In-depth exploration into the multifaceted regulatory mechanisms of carotenoid metabolism in microalgae. Bioresour. Technol..

[159] Patel A.K. (2025). Microalgae in Dual Role: An Effective Platform for Biological Carbon Capturing and High-Value Carotenoid Production. Curr. Pollut. Rep..

[160] Hanifa N.M., Thevarajah B., Nimarshana P.H.V., Perera M.A.R.C., Harshan G.M.M., Abeyarathna D.R.P., Ariyadasa T.U. (2025). Starch Wastewater-integrated Microalgae Cultivation: A Circular Bioeconomy Approach for Bioremediation and Natural Carotenoids Production. ACS ES T Water.

[161] Dodangodage C.A., Gamage G.N., Wijesekara I.A., Kasturiarachchi J.C., Perera T.A., Rajapakshe D., Halwatura R.U. (2026). Valorization of Canteen Wastewater Through Optimized Spirulina Platensis Cultivation for Enhanced Carotenoid Production and Nutrient Removal. Phycology.

[162] Braga M.Q., de Assis L.R., Ferreira J., Ribeiro V.J., Calijuri M.L., Assemany P.P. (2023). Microalgae cultivation and carotenoid production in paint booth effluent mixed with domestic sewage. Int. J. Environ. Sci. Technol..

[163] Guajardo-Barbosa C., Guajardo-Rodríguez T., López-Chuken U.J., Barceló-Quintal I.D., Cruz-Chávez D., Beltrán-Rocha J.C. (2025). Adaptation of Microalgae for the Production of Settling Flocs, Carotenoids, and Mineral Recovery from Municipal Secondary Effluents. Phycology.

[164] Elango A., Goel M., Kumar G., V P C., Priya V V., Ngamcharussrivichai C., G F., Ashokkumar V. (2026). Biochar production with concomitant biopolymer accumulation of microalgae-based wastewater treatment for carbon neutrality. DeCarbon.

[165] Huang L., Liu L., Zhang G., Li G., Fang Y., Li Y., Yang H., Xu H. (2026). A membrane-mediated algal-bacterial coupling strategy for energy—Efficient and low-carbon PHA production. Bioresour. Technol..

[166] Restiawaty E., Salman N.S., Maria R., Wibisono T.A.S.E., Budhi Y.W. (2025). Integrating biopolymer production and wastewater treatment: Sustainable cultivation of Porphyridium cruentum on industrial waste streams. Chem. Eng. J..

[167] da Silva G.d.A., João J.J., Sales R.d.O.J., Becker D., Skoronski E., Neves F.d.F. (2024). Utilizing Parachlorella microalgae and Arthrospira cyanobacteria for tertiary wastewater treatment and biomass valorization as raw material for biopolymer production. Clean Technol. Environ. Policy.

[168] Machado G.d.O., De Assis M.L., Reis M.F.d.C., Alexandre M.A.d.S., Arruda T.R., Pereira A.S.A.d.P., Calijuri M.L., de Carvalho J.M.F., Carneiro A.d.C.O., Jesus M. (2026). Microalgae-Derived Biopolymers: An Ecological Approach to Reducing Polylactic Acid Dependence. Sustainability.

[169] Arashiro L.T., Josa I., Ferrer I., Van Hulle S.W.H., Rousseau D.P.L., Garfí M. (2022). Life cycle assessment of microalgae systems for wastewater treatment and bioproducts recovery: Natural pigments, biofertilizer and biogas. Sci. Total Environ..

[170] Alazaiza M.Y.D., Albahnasawi A., Ahmad Z., Bashir M.J.K., Al-Wahaibi T., Abujazar M.S.S., Abu Amr S.S., Nassani D.E. (2022). Potential use of algae for the bioremediation of different types of wastewater and contaminants: Production of bioproducts and biofuel for green circular economy. J. Environ. Manag..

[171] Bhatt A., Khanchandani M., Rana M., Prajapati S.K. (2022). Techno-economic analysis of microalgae cultivation for commercial sustainability: A state-of-the-art review. J. Clean. Prod..

[172] Barnharst T., Rajendran A., Sun X., Hu B. (2023). Process optimization of aquaculture wastewater treatment using a mycoalgae biofilm. Algal Res..

[173] Maciel de Castro A.P., Quintão Braga M., Viana Araujo P., Carvalho Nogueira da Gama R., Saleme Aona de Paula Pereira A., Abrantes Silva T., Silva Henriques B., Ferreira Lorentz J., Vilela Avelar N., Calijuri M.L. (2026). Emerging trends in wastewater-grown microalgae: A techno-economic and environmental perspective. Biomass Bioenergy.

[174] Silva Machado R.L., Deprá M.C., Dutra D.A., Schneider A.T., Machado E.F., Zepka L.Q., Jacob-Lopes E. (2026). Why Does Microalgae Biodiesel Not Work?. Processes.

[175] Dias R.R., Deprá M.C., de Menezes C.R., Zepka L.Q., Jacob-Lopes E. (2025). Microalgae Cultivation in Wastewater: How Realistic Is This Approach for Value-Added Product Production?. Processes.

[176] Bisht B., James J., Ahmad W., Kumar A., Dmitriev A.A., Pal M., Huang J., Vlaskin M.S., Verma M., Kumar V. (2025). Exploring microbial electrochemical technology for the treatment of wastewater and removal of ampicillin. Chem. Eng. J..

[177] Aletheia S., Syauqi A., Kelvin, Khaira K., Rafi M. (2024). Techno-economic analysis of biodiesel and bioethanol production from *Chlorella* sp. algae biomass. E3S Web Conf..

[178] Alprol A.E., Özkaya B., Ali M., Zaidi A.A., Naseer M.N. (2026). Chapter 18—Techno-economic analysis of microalgal biodiesel produced by conventional and renewable energy sources. Sustainable Production of Microalgae Biomass as a Biodiesel Feedstock.

[179] Jena A., Thomas A.P., Samal B.B., Dubey B.K., Kumar C.S., Varshney S.K. (2025). Assessing the anaerobic biodegradability of PHB filament and its 3D printed sheets through biochemical methane potential (BMP) test. Sci. Total Environ..

[180] Rafiq A., Morris C., Schudel A., Gheewala S. (2025). Life Cycle Assessment of Microalgae-Based Products for Carbon Dioxide Utilization in Thailand: Biofertilizer, Fish Feed, and Biodiesel. F1000Research.

[181] Hernández A., González-Moya M., Márquez A., Acevedo L. (2024). Review microalgae drying: A comprehensive exploration from conventional air drying to microwave drying methods. Future Foods.

[182] Chaurasiya S., Preuss N., You F. (2026). Life cycle assessment of seaweed-based biorefineries: Environmental impacts, hotspots, and pathways for a circular bioeconomy. RSC Sustain..

[183] Laouane H., El Joumri L., Halhaly A., Arid Y., Labjar N., El Hajjaji S. (2026). Life-Cycle Assessment of Wastewater Treatment: Enhancing Sustainability Through Process Optimization. Sustainability.

[184] Dewalkar S.V., Shastri S.S. (2022). Integrated life cycle assessment and life cycle cost assessment based fuzzy multi-criteria decision-making approach for selection of appropriate wastewater treatment system. J. Water Process Eng..

[185] Vázquez-Romero B., Perales J.A., Pereira H., Barbosa M., Ruiz J. (2022). Techno-economic assessment of microalgae production, harvesting and drying for food, feed, cosmetics, and agriculture. Sci. Total Environ..

[186] Badruddin I.J., Gautam A., Heer K., Bind A., Goswami L., Kushwaha A., Kim B.S., Bhan U., Hussain C.M., Kushwaha A., Bharagava R.N., Goswami L. (2023). Chapter 19—Upstream and downstream processing of microalgae-based processes for simultaneous wastewater treatment and pigment production. Bio-Based Materials and Waste for Energy Generation and Resource Management.

[187] Ferreira J., Braga M.Q., Gama R.C.N.d., Magalhães I.B., Marangon B.B., Castro J.d.S., Lorentz J.F., Henriques B.S., Pereira A.S.A.d.P., Assemany P.P. (2024). Carotenoids from wastewater-grown microalgae biomass: Life cycle assessment and techno-economical analysis. J. Clean. Prod..

[188] Mondal S.K., Kar S.P., Mitra R., Maity A., Shukla A., Tribedi P., Gupta A.D. (2026). Bio-aggregate Dynamics: A Key Driving Force for Microalgae Doped Wastewater Treatment Technology Within Single Chambered Photobioreactor. Waste Biomass Valorization.

[189] Kumar B., Ghosh T., Purewal S.S., Bala K. (2023). Life Cycle Assessment (LCA), Techno-Economic Analysis (TEA) and Environmental Impact Assessment (EIA) of Algal Biorefinery. Algae Refinery.

[190] Korsah M.A., Gyau T., Lyon D., Danquah M.K., Gagnon Y., Jacob-Lopes E., Zepka L.Q., Deprá M.C. (2025). Chapter 22—Techno-economic analysis of downstream processing of microalgae. Algal Bioreactors.

[191] Olymon K., Sabari R., Roy N., Bhattacharjee I., Sharma N., Ahmed S., Shukla J., Kumar A. (2026). Sustainable Biorefineries: Integrating Techno-Economic Analysis and Life Cycle Assessment in the Circular Bioeconomy.

[192] Kumar N., Banerjee C., Chang J.-S., Shukla P. (2022). Valorization of wastewater through microalgae as a prospect for generation of biofuel and high-value products. J. Clean. Prod..

[193] Pereira A.S.A.d.P., Silva T.A., Magalhães I.B., Ferreira J., Braga M.Q., Lorentz J.F., Assemany P.P., Couto E.d.A.d., Calijuri M.L. (2024). Biocompounds from wastewater-grown microalgae: A review of emerging cultivation and harvesting technologies. Sci. Total Environ..

[194] Biliani S.E., Manariotis I.D. (2026). High-rate algal ponds in wastewater treatment: A critical look at recent developments. Environ. Sci. Water Res. Technol..

[195] Chandola G., Muthuraj M., Mandal S., Vismaya P.S., Bhunia B. (2026). Engineering advances in tubular photobioreactors for microalgae cultivation: Design, scale-up, optimization, and operational challenges. Renew. Sustain. Energy Rev..

[196] Hawrot-Paw M., Sąsiadek M. (2023). Optimization of Microalgal Biomass Production in Vertical Tubular Photobioreactors. Energies.

[197] Ukogo I., Ifeanyi V., Ekwenye U. (2026). Overview of the Role of Microalgae in Wastewater Management: Challenges and Opportunities. Int. J. Res. Innov. Appl. Sci..

[198] Mpongwana N., Kumari S., Rawat I., Zungu P.V., Bux F. (2026). Resource recovery from dairy wastewater for circular-economy: A review of opportunities, challenges, and prospects. Environ. Dev. Sustain..

[199] Xu P., Shao S., Qian J., Li J., Xu R., Liu J. (2024). Scale-up of microalgal systems for decarbonization and bioproducts: Challenges and opportunities. Bioresour. Technol..

[200] McGrath S., Laamanen C., Senhorinho G., Scott J. (2023). Microalgal harvesting for biofuels—Options and associated operational costs. Algal Res..

[201] Crippa I., Dolci G., Grosso M., Rigamonti L. (2024). Life Cycle Assessment of Microalgal Biomass Valorization from a Wastewater Treatment Process. Waste Biomass Valorization.

[202] Choudhary S., Venkatrayalu G., Ravi Kiran B., Poluri K.M. (2025). Advances and Perspectives of Microalgal Biorefineries for Bioenergy. Energy Fuels.

[203] Verma A., Saini G., Naushad M., Mohamed Kutty S.R., Hossain M.S., Birniwa A.H., Jagaba A.H. (2025). Chapter 20—Techno-economic and lifecycle assessment of industrial effluent treatment in biorefineries. Biorefinery of Industrial Effluents for a Sustainable Circular Economy.

[204] Fitriyah I.J., Saputro S., Sajidan S. (2026). Wastewater-to-biofuels and bioproducts through integrated anaerobic digestion, bioelectrochemical systems, and algal biorefineries: A systematic review of techno-economic and life-cycle evidence. Biotechnol. Biofuels Bioprod..

[205] Fasaei F., Bitter J.H., Slegers P.M., Van Boxtel A.J. (2018). Techno-economic evaluation of microalgae harvesting and dewatering systems. Algal Res..

[206] Budiarto A., Hardianto A., Syihab A., Sidquni S., Adinisa L., Nurjannah I., Subroto T. (2026). Comparison of Microalgae Harvesting Methods: Technical Efficiency and Economic Feasibility for Scalable Biofuel Production. Trends Sci..

[207] Dębowski M., Kisielewska M., Zieliński M., Kazimierowicz J. (2026). Anaerobic Digestion of Microalgal–Bacterial Consortia Biomass: Challenges and Prospects for Circular Wastewater Treatment. Appl. Sci..

[208] Pérez Mesa A., Céspedes Grattz P.A., Vidal Vargas J.J., Ríos L.A., Ocampo Echeverri D. (2025). Techno-Economic Assessment of Microalgae-Based Biofertilizer Production from Municipal Wastewater Using *Scenedesmus* sp.. Water.

[209] Nobre M.L.F., Tavares D., Fraga C., Oliveira B., Dias M., Mesquita S., Oliveira C.M., Pires J.C.M. (2024). Techno-economic analysis of a circular microalgal approach for enhanced wastewater treatment and resource recovery in Northern Portugal. J. Clean. Prod..

[210] Ferreira F., Ortigueira J., Reis A., Lopes T.F. (2025). Benchmarking commercially available value-added fractions with potential for production via microalgae-based biorefineries: Is it worth it?. Biotechnol. Biofuels Bioprod..

